# Supplementary material for: A high-content RNAi screen reveals multiple roles for long noncoding RNAs in cell division
Source: Nat Commun. 2020 Apr 15;11:1851. doi: 10.1038/s41467-020-14978-7 (PMC7160116; doi:10.1038/s41467-020-14978-7)
Supplement: Supplementary file 1 — Supplementary Information [file 41467_2020_14978_MOESM1_ESM.pdf]

Supplementary Information

"A high-content RNAi screen reveals multiple roles for long noncoding RNAs in cell division" by L. Stojic et al.

The PDF file includes:

Supplementary methods: sequences of *linc00899* and *C1QTNF1-AS1* vectors (Labomics)

Supplementary tables (10)

Supplementary figures (15)

Supplementary Methods

LncRNA sequence from *Labomics*

>*Linc00899*_full sequence

cggccgcccccgaagcgctgctgtcaccccggccgcgccccccaactttctgcacagtcgcggagctggaagtttccggg

cttcgcggacacgctgggctgggtttcagtcgcggctccgaggttggcaacaaagagggaaagaaggaggaaaagcaggc

cggggaggggaggaagagaaccgcgcggaggccgcggcgccgagagccccagaacttccaattctacccagaagcttttt

tcgtcgtgtttttctcttagacatgatcctctctgaggttggtcctgggcttccatacgtgattcatggaagaggtctca

gccccaagagcccctgagggtactgtccactccccctggaaacttccagaacctgacgtggggctgaagacatagaggct

ctgagagttacataattgattctgactttggctgttggtcaacagtgtcataaggtaaaataaggctgttgtagaatctg

ctcagccagggaggggaaaggagtcggcaatcaggtctcctcctgggcacctttgtgaggccagctggcgagagtggggg

gtgacactgaggtcccagcagctccaaatgcaggcagagccctgtcctcagagaaggtcacagcctagccaagcccagcc

aggtggatgggcccacggaacgcacaggaacctggaacggaggttgaaagcaggaagcacagtctgtgactccccagccc

actctgcattcgaccacttggggcccagaagcttcaggaaaggtgcacaaggtcactgggtcccagtactcccaacagga

aggtctggtccagggacagggctcttcccgactccccttagccacacgcaccagaagttctgcagtgcccagtgggcata

gcagtccccaagaatgacccagcactgaagctgagccaaagaacttggggagcgagccacacccccctcactccctcttcg

cctgctccagacttgccaggcggttgcttttcgtgggaaccgggatgtcctcaccaccctgtccagggcccagccccatg

tccctggcctgctacagctggaaaaaaaaaagagagatgtttgtttttatttgtttataaaaagaaaagtgttatatata

taacatattatacctcatgaatacatacaattatttgtcaattaacaataaagaaaaatacagcaagcaaaaaagactct

cttccacaaaaatagtgttcattacagaaaagtacaaaaaaaaaaaaaaaaaaactaagaggatatttagaattaagaaaa

aactaagagggtatttagaattaaaaaataaaaagaaaacaattacccatgaggtaattactgaatgcatttggttgaaa

gtccttctgctatattttccaaactgtatgtgtatatatgtgcgcatgcatatatgtgtttgtgtgtgtgtacacatatc

tatatgaatggaccatatcgtaagttataaatgcatacatatattcatgtatatataatcattagatcatactataggtt

attttacagtctttttgttgaatacgttgtgagcattttatgtcattattttctacaagatttgaaaaataaagtataaa

taccagttaa

>*Linc00899*_scrambled sequence

ccagctctgaagaacagatgccatgttttaatatctcaagtcggagaaaatatttcgctgggtgcgaagtacttagcat

agatggctccctaggtcattgacatgttctgagacgcttagccaaagtctccatgtgacgtcatatcacaaccacccag

cagcgaagacatgcaaatagatatatgagacgcctcgggtctttcacacaccgtatacctggtcataatctgctccttt

acatcgcgtacttcttgtgtcagggtcttgggggacctttcggaatcatatgtagccggtgtcagacgaaaatgcgtta

tccatcttggcacgtggcgcagtcacctcaagtaagtgcagacaatggttcctatacctttcgtgcacggttgtaataa

tcaaggatggcgttactgttggcaggcaccctatttagccccacgcctcggtctagctggtgcaagctgggtattgtca

acatgctagaaaagtataaagaaccagtgtcacatttttcgaagcactcattggaaagagaaacatcttgcgtctcttt

gatatttaatgtaaatggaattgctccatcttacttagaaccgaaagctcagtaaagaaaacactcggggcgcctattg

cgcgtactgaaaaaccgaactgcagtcatcgtggaacagggagaccccaaccgacaataccacaggccaaaggccgtac

tgagtcttcgacagttataattgaaaacaatcaactcttcgcgatgataagaaaagttaccccaggattatgtgtggct

ctgagaaagaagaggcggcaagtgctaagaattatatactcggtgacggaagcaacggagtccgtatgcagttacagct

tcgtcgataaggtttgcgtggatccatggctcccgctaggtgttgttcgcgtccgggcaggcagtgcacaaaaggagcc

tgaactactgtcagccgaatgactgcggaaaaaatcgttagaaacatcgacataagtgcggcaaatcacagttgcaatc

aggcagtccacgtgtagaatatctcacacaataaggcgcatgcttcccataaatgtattgagaattagcctacatccgg

taggcgtcaacctagtagcgttgatgatcggcagatcacggtgtccgtcgccctcgggtcgtcgtcactacctatacat

acaccacatatacaatggggataaagaaaccagaaaggctatctagaatttgggacatgaacagtgcgttaatccaaca

cacacagggataagacattccgaacgcgttttcttacgatcgttgataaatgagagggatcgccattcaccagttgaga

acactgaccccgtcatgagtatcattgaagtacaaaattggaccaagcggacgatttcatgaagtacagtggaccccccg

atactcgacattccagtttgaaaagaggtatgtccgcgtaaggactttggggcacgacaccaggatatcatatcgcgcg

gactagttgcaacttacctgcgagggtagcgggcatagacccagagcttctggacggccttcagtggttaggagtacac

gggaaaggcaccccagactgaacgcattga

>*C1QTNF1-AS1*_full sequence

gaaggaggaaaggagtgagcatgtcctgctcctgcatgtccctgcttaagctcaggactggcccttccaggccaaggac

cccagcatagaccccaggacagggccccaaggatccctggctcatgagagcggcttgctgggctgccccaagagagcct

gaaggaaacacattgttgagctgagctgacgtcgctgtttcttccagactgctctctaaagtgggcagggtagcgaccg

gccggctccgatggtgacgtcccactgccaaggggtgggagtggggagagtctccacagagcttcggagaagctgctaa

gatggaaaagtggaaacttggcagacagatccagcctccctggccactggcccatgctcgtggctcctggatggcgctg

ccacgttctgagcagcttgggacaggtggagatcaggactggcagctgcaaggacacaccagagccacagaaactaaag

agaatttccaaaaggagtctatggtgaagtctctgaggatgcaaagaagacaaggagaatgaaaatccaatgaaagcct

gattgtatttgttgaccttaaggaaagtgattttatggtacagcctctctggaagggagggtgtgttcgctcacagaat

gcaaataccctttgaccccctaatctttcttctaggagtttctcctacagataaacttagaagggtgctcaaataagta

agttcaaggatatcctctgaagcattgccgtagtataaaaaaagcacagataccctcaaaggacatcattaggggcctg

gtaaaataaattccacacagtggaacaccgtgtagctctttagagaataaacagctctctatatgtgatctggaa

>*C1QTNF1-AS1*_scrambled sequence

gtggcggatgttggcttagaatcaggattgtgcagatattgaaccttgttgcctcccaccctgcacagcttgatgaccc

ctaaaagtagcagagcttcacaagtactattgggcaatcgctccggcgatggtgaaaaaacctactcttcaacacggtc

gagcttggtctaccctcttcagcgaacattacaagaaacagaactcataggcatcaaatgtttgaggaccatggaggga

tatgcctagacgcgcggtagcaggcaagatcagggtttaacatggcctcttgaaacttagaggtacagccgccgcgcgt

ccgcggtagtgcactgcggaaaccgcataatatcgtgaggtaggtgggcgaagtggtcttggtgcagcttgaccttaag

tgttaggacccatgtagatagcaaaagcagggcggcggcgtcataaagggttcagcgcacccatgcgaggaagttagga

gagaaccaaggacaatcgggccatcaggatgacaggcagaaacgaagtacacacagaagcgatccgtttattaacgcaa

cattggcggctatcaacacattcgtcctattgtgtaggttgcatggtaattctgtcatataaggcgtgtccccaacaca

ataaaggttcgccgctaagctcgccaaatccgatcaggcaacaaagtcggatctatactagtctaaagaggcggagagg

ctagagcttcaatccgccaccgcgaccaattctaagttaggttagtgtcgactgtgaaagcaaagaccggcctcaacgg

gtagtgagcggggatggactggagtcctaagcacacgctgttgcgctagagaatctaggactcggttactcatcc

Expression primers	Forward primer (5'to3')	Reverse primer (5'to3')
GAPDH	CAACAGCCTCAAGATCATC AG	ATGGACTGTGGTCATGAGTC
RPS18	ATCCCTGAAAAGTTCCAGC A	CCCTCTTGGTGAGGTCAATG
ACTB (β-actin)	GTTACACCCTTTCTTGACA AA	GTCACCTTCACCGTTCCAGT T
NORAD	AGCGAAGTCCCGAACGAC GA	TGGGCATTTCCAACGGGCCA A
MALAT1	GACGGAGGTTGAGA TGAAGC	ATTCGGGGCTCTGT AGTCCT
U1	ATACTTACCTGGCAGGGG AG	CAGGGGGAAAGCGCGAACG CA
Linc00899/LOC100271 722 (exon 1-2)	gagagccccagaacttccaa	agcctctatgtcttcagccc
Linc00899/LOC100271 722 (exon 2-4)	ctgagggtactgtccactcc	ccctggctgagcagattcta
C1QTNF1-AS1/LOC100507410 (exon 1-2)	agctgacgtcgctgtttctt	tccgaagctctgtggagact
C1QTNF1-AS1/LOC100507410 (exon 2-3)	ggacaggtggagatcaggac	tctccttgtcttctttgcatcc
DUBR/LINC00883 (QIAGEN)	/	QT02433235
PP7080/ LOC25845 *(QIAGEN)*	/	QT01159095

LOC100506835/linc00840 (exon 1-2)	ttgcgcacgaattttacac	gtggaccttgtctgcatcct
LOC729970 (exon 2-3)	gaaccacaaagctgaacctca	agttctgagagcacaagggg
TPPP (QIAGEN)	/	QT00007693
CCNB1	TTTGCACTTCCTTCGGAGAGC	AAGGAGGAAAGTGCACCATGTC
RAI14		QT00050470
DNAAF5/HEATR2		QT00093765
ITGB1BP1		QT00024654

Supplementary Table 1. List of primer sequences for qPCR

siRNA	Sequence (antisense)	Product number
siGENOME Non-Targeting siRNA Pool #2 (GE Dharmacon)	/	001206- 14- 20
Negative control siRNA #1 (Thermo Fischer Scientific, Silencer select, Ambion)	/	4390084
GNG12-AS1 exon 1 siRNA #1 (Life Technologies, Silencer select) #S59962	AUUCUUGUUCACGUCGCCG	4392421
Lincode Human Linc00899 SMART pool, GE Dharmacon	CAGAGAAGGTCACAGCCTA GGGGAAAGGAGTCGGCAAT GGACCATATCGTAAGTTAT GACTTTGGCTGTTGGTCAA	R-189507-00-0005
Lincode Human C1QTNF1-AS1 SMART pool, GE Dharmacon	AGATAAACTTAGAAGGGTG ACUAGAGGCTGCAGCGGCA TCTATATGTGATCTGGAAA TCATTAGGGGCCTGGTAAA	R-189493-00-0005
Lincode Human Linc00883/DUBR SMART pool, GE Dharmacon	/	R-023569-00-0005
Lincode Human PP7080 SMART pool, GE Dharmacon	/	R-028371-00-0005

Lincode Human Linc00840 SMART pool, Dharmacon	/	R-189275-00-0005
Lincode Human LOC729970 SMART pool, Dharmacon	/	R-182791-00-0005
Lincode Human NORAD *SMART pool, Dharmacon*	/	R-038095-00-0005
TPPP SMARTpool: ON-TARGETplus (GE Dharmacon)	/	L-019695-01-0005
Ch-TOG/CKAP5 SMARTpool: ON-TARGETplus (GE Dharmacon	/	L-006847-00-0005
ECT2 SMARTpool: ON-TARGETplus (GE Dharmacon	/	L-006450-00-0005

Supplementary Table 2. List of siRNA sequences

LNA	Sequence (5’ to 3’)	Product number
Negative control LNA A	AACACGTCTATACGC	300611-00
Negative control LNA B	GCTCCCTTCAATCCAA	300615-00
Linc00899 LNA gapmer_1 (exon 1)	cgcgactgtgcagaa	300603-00<br>Design ID:606267-2
Linc00899 LNA gapmer_2 (exon 4)	acttctggtgcgtgtg	300603-00<br>Design ID:380384-2
Linc00899 LNA gapmer_3 (exon 1)	cgactgtgcagaaagt	339511<br>LG00204910-DDA
Linc00899 LNA gapmer_1 (intron 1) #4916	ctggtgaggaagaaca	339511<br>LG00204916-DDA
Linc00899 LNA gapmer_1 (intron 2) #4909	tagtgtatgatagaac	339511<br>LG00204909-DDA
Linc00899 LNA gapmer_1 (intron 3) #4912	acatagaatctggaat	339511<br>LG00204912-DDA
C1QTNF1-AS1, LNA gapmer_1 (exon 3)	tacggcaatgcttcag	300603-00<br>Design ID:591242-1
C1QTNF1-AS1, LNA gapmer_2 (exon 2)	gctcagctcaacaatg	300603-00<br>Design ID:591242-2

Supplementary Table 3. List of LNA Gapmer sequences (ordered from Exiqon)

Antibody	Usage	Dilution	Catalog number
TPPP	Western blot	1-1000	NBP2-34031, Novus
β-tubulin	Western blot	1-2000	T019, Sigma
p150	Western blot	1-2000	610473, BD Transduction Laboratories
Cyclin B1	Western blot	1-1000	12231S, Cell signalling
RAI14	Western blot	1-300	NBP1-94075, Novus Biologicals
DNAAF5/HEATR2	Western blot	1-250	HPA020243, Atlas Antibodies
ITGB1BP1	Western blot	1-250	HPA071538, Atlas Antibodies
Acetylated α-tubulin	IF	1-500	T6793, Sigma
EB1	IF	1-300	CRUK Hybridoma bank *(Zyss et al., 2011, JCB)*
α-tubulin	Imaging screen, IF	1-1000	TUB9026, Sigma
Anti-centromere protein (CREST)	IF	1-1000	15-234-0001, Antibodies Inc
Mad2	IF	1-100	924601, Biolegend (Babco, Poly19246)
Phospho Histone H3 (Serine 10)	Imaging screen, IF<br>Western blot	1-2000<br>1-1000	06-570, Millipore
α-tubulin (rat)	Imaging screen, IF	1-500	MCA78G, AbD Serotec
γ-tubulin	Imaging screen, IF	1-1000	GTU88, Sigma
CDK5RAP2/CEP215	Imaging screen	1-500	(Barr et al., 2010, JCB)
H3K4me3	CUT&RUN	1-100	05-1339, Millipore (LOT2780484)
H3K36me3	CUT&RUN	1-100	61101, Active Motif (LOT32412003)
H3K27me3	CUT&RUN	1-100	9733S, C36B11, Cell Signaling (LOT8)
H3K27ac	CUT&RUN	1-100	Ab4729, Abcam (LOT GR3187598-1)

Goat anti-rabbit IgG secondary antibody	CUT&RUN	1-100	Ab97047, Abcam (LOT GR254157-8)
Rabbit anti-mouse IgG secondary antibody	CUT&RUN	1-100	A27022, Thermo Fisher Scientific (LOT RG240909)
Alexa Fluor® 555 goat anti-rat	Imaging screen, IF	1-1000	A-21434, Thermo Fisher Scientific
Alexa Fluor® 488 donkey anti-rabbit	Imaging screen, IF	1-1000	A-21206, Thermo Fisher Scientific
Alexa Fluor® 647 donkey anti-mouse	Imaging screen, IF	1-1000	A-31571, Thermo Fisher Scientific
Alexa Fluor® 647 goat anti-human	IF	1-1000	A-21445, Thermo Fisher Scientific
Amersham ECL anti-mouse IgG, HRP antibody	Western blot	1-2000	NXA931V, GE Healthcare
Amersham ECL anti-rabbit IgG, HRP antibody	Western blot	1-2000	NA934V, GE Healthcare
Alexa Fluor 568 Phalloidin	Imaging screen	1-500	A12380, Thermo Fisher Scientific

Supplementary Table 4. List of antibodies

CHART probes	Probe (5'to3')
*Linc00899*_CHARTprobe1_exon 1	cgcgactgtgcagaaagttggggg/iSp18/3BioTEG
*Linc00899*_CHARTprobe14_exon 1	cgaagcccggaaacttccagctcc/iSp18/3BioTEG
*Linc00899*_CHARTprobe9_exon 4	ccccaagttctttggctcagcttc/iSp18/3BioTEG
*Linc00899*_CHARTprobe10_exon 4	tggacagggtggtgaggacatccc/iSp18/3BioTEG
*Linc00899*_CHARTprobe10_exon 4_ sense (negative control)	gggatgtcctcaccaccctgtcca/iSp18/3BioTEG
*Linc00899*_CHARTprobe14_exon 1_ sense (negative control)	ggagctggaagtttccgggcttcg/iSp18/3BioTEG

Supplementary Table 5. List of CHART probes (ordered from ATDBio Ltd, School of Chemistry, Southampton, UK)

CHART primers	Forward primer (5'to3')	Reverse primer (5'to3')
*Linc00899*_exon 4	agtccccaagaatgacccag	gacagggtggtgaggacatc
*Linc00899*_exon 1	ccccaactttctgcacagtc	ccctctttgttgccaacctc
5.8 S (West et al., 2014)	GGTGGATCACTCGGCTCGT	GCAAGTGCGTTCGAAGTGTC

Supplementary Table 6. List of CHART primers

Targeted lncRNA	Guide-ID	Guide sequence
C1QTNF1-AS1_guide 70 Forward (5'-3') Cloned into Addgene vector #48138	Guide 70 (112bp from TSS)	caccGTCCCTGCTTAAGCTCAGGAC
C1QTNF1-AS1_guide 70 Reverse (5'-3') Cloned into Addgene vector #48138	Guide 70 (112bp from TSS)	aaacGTCCTGAGCTTAAGCAGGGAC
SSODN_ C1QTNF1-AS1_ guide 70 (3'-5')	Guide 70	GCAAGCCGCTCTCATGAGCCAGGGATCCTTGGGGCCCTGTCCTGGGGTCTATGCTGGGGTCCTTGGCCTGGAAGGG*cacacaaaaaaccaacacacagatctaatgaaaataaagatcttttatt*GTCCTGAGCTTAAGCAGGGACATGCAGGAGCAGGACATGCTCACTCCTTTCCTCCTTCCCGAGCCCCGCAGTGAG

Supplementary Table 7. List of CRISPR pAS guide RNA sequences and single stranded DNA donors (SSODN) containing SV40 polyA sequence (*in italic*)

Targeted lncRNA	Forward primer (5'to3')	Reverse primer (5'to3')	PCR product size
C1QTNF1-AS1_Guide 70	tcccttcctgctcacaccgc	aggaccctgccatctccctga	Wild type=358bp, pAS inserted=404bp, cleaved PCR product after BglII should give rise to 2 bends, 149bp and 237bp.
Forward primer for Addgene vector #48138	CATGATTCCTTCATATTTGCATATAGC		(used for Sanger sequencing)
mU6	GAGATCCAGTTTGGTTAGTACCGGG		(used for Sanger sequencing of CRISPRa clones)

Supplementary Table 8. List of PCR primers for CRISPR pAS insertion

Targeted lncRNA	Guide-ID	Guide sequence
Linc00899	Guide 1 (-129bp from TSS)	AGATCGTCCCGAGCGCGCCG
Linc00899	Guide 2 (+20bp from TSS)	GGGTGACAGCAGCGCTTCGG
Negative control	sgRNA 2	GTGCGATGGGGGGGTGGG TAGC

Supplementary Table 9. List of CRISPRa guide RNA sequences cloned into Addgene vector #60955

Targeted lncRNA	Forward primer (5'to3')	Reverse primer (5'to3')
Linc00899 full	GGGGACAAGTTTGTACAAAAAAGCAGGCTcggccgcccccgaagcgctgctgtc	GGGGACCACTTTGTACAAGAAAGCTGGGTttaactggtatttatactttatttt
Linc00899 scr	GGGGACAAGTTTGTACAAAAAAGCAGGCTccagctctgaagaacagatgccatg	GGGGACCACTTTGTACAAGAAAGCTGGGTtcaatgcgttcagtctggggtgcct

Supplementary Table 10. List of PCR primers used for subcloning of *linc00899* (full and scrambled sequence obtained from the Labomics) into the lincXpress vector with the Gateway system

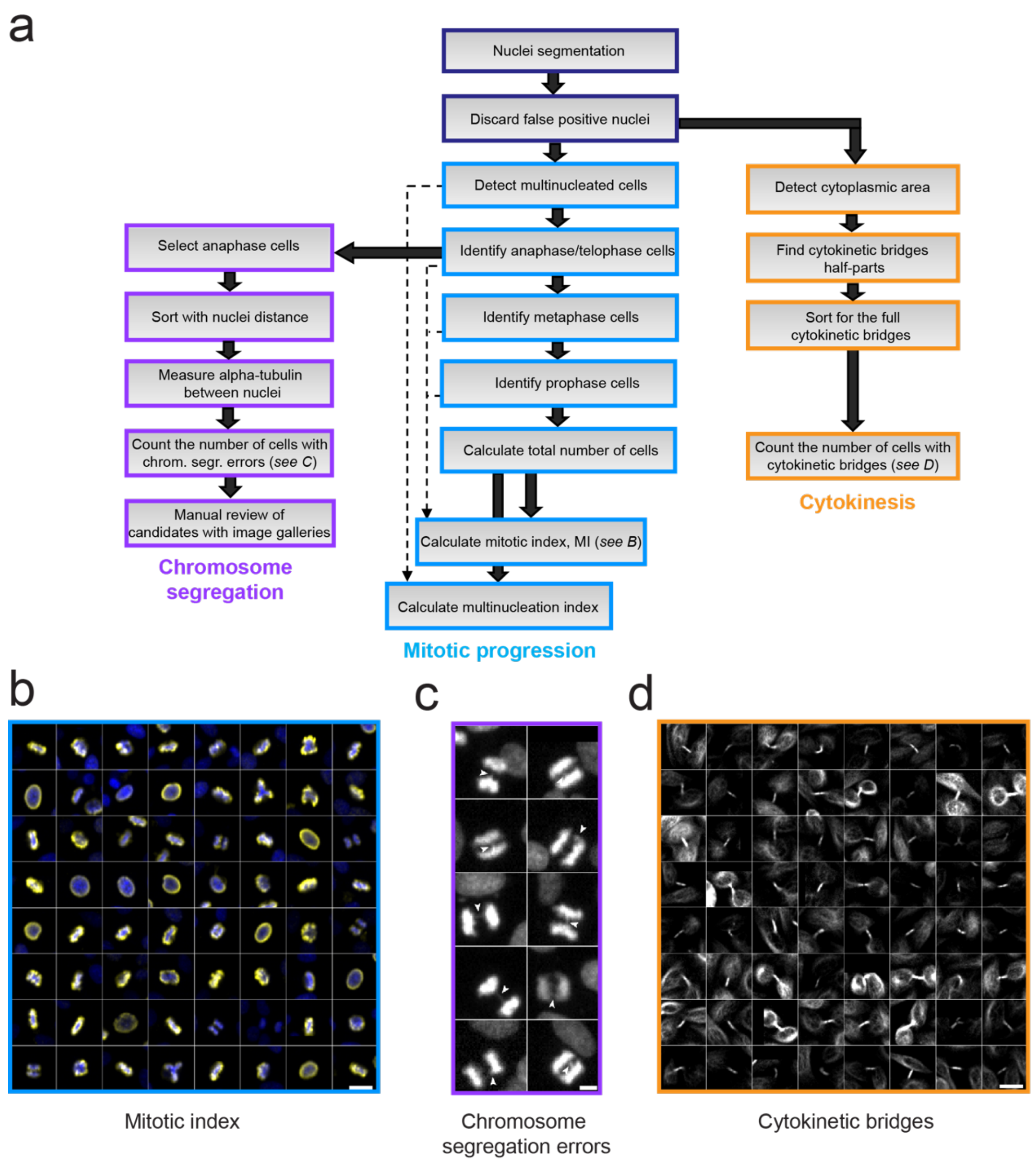

Supplementary Figure 1. Workflow to identify new lncRNAs in regulation of cell division.

a. Schematic of the workflow for automated cell segmentation and quantification of cellular features after depletion of each lncRNA in the Lincode library. This includes the number of mitotic cells (i.e., the mitotic index), the number of chromosome segregation defects in anaphase cells and the number of cytokinesis defects.

b. Gallery of representative images of mitotic cells positive for PHH3 staining (yellow). DNA is stained with Hoechst and is shown in blue. Scale bar, 20 µm.

c. Gallery of representative images of cells with chromosome segregation defects. Arrows mark chromosome segregation errors. Scale bar, 20 µm.

d. Gallery of representative images of cells with cytokinetic bridges. Scale bar, 20 µm.

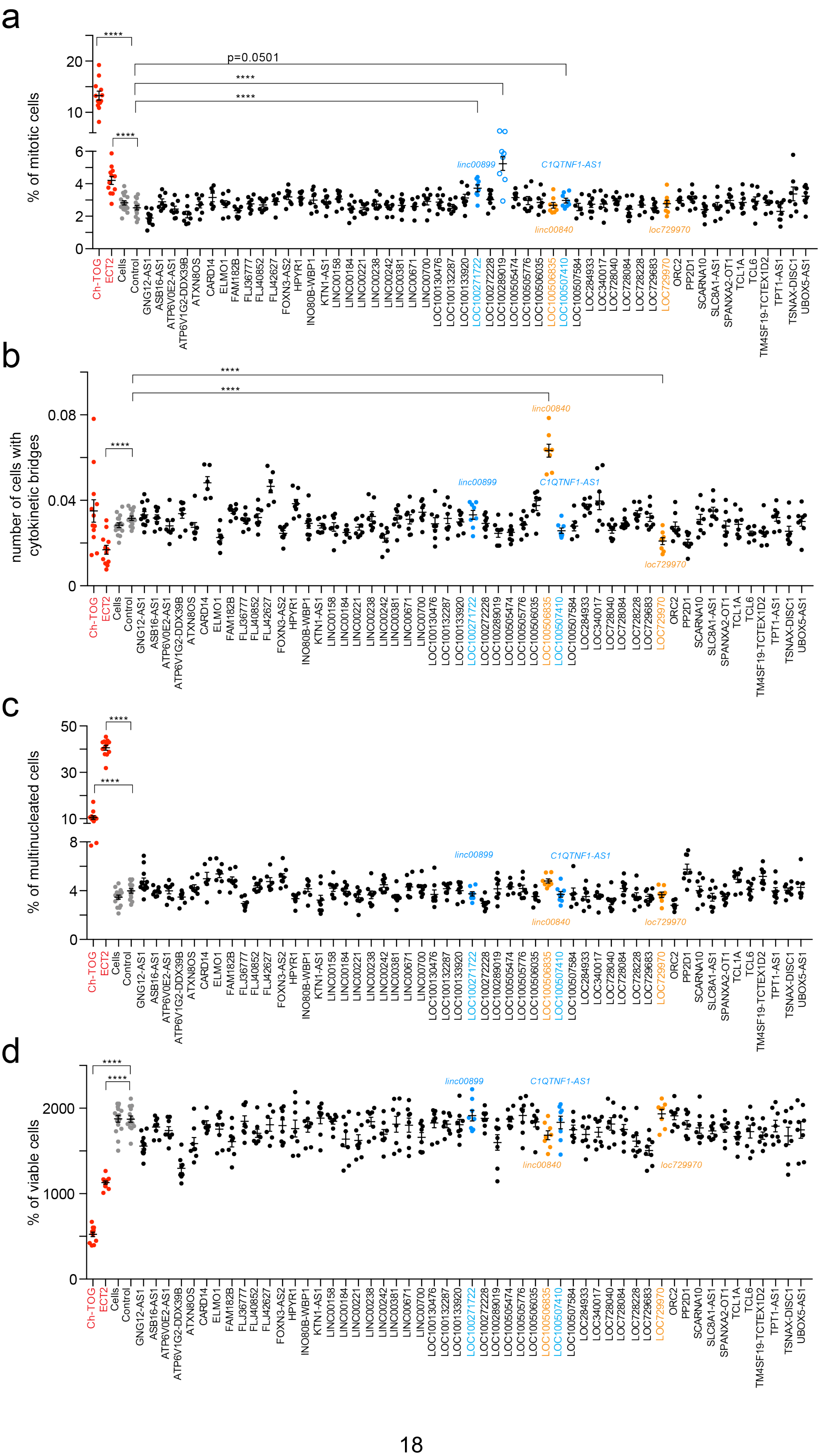
a
% of mitotic cells
20
10
6
4
2
0
****
p=0.0501
****
****
****
linc00899
C1QTNF1-AS1
linc00840
loc729970
Ch-TOG ECT2 Cells Control GNG12-AS1 ASB16-AS1 ATP6V0E2-AS1 ATP6V1G2-DDX39B ATXN8OS CARD14 ELMO1 FAM182B FLJ36777 FLJ40852 FLJ42627 FOXN3-AS2 HPYR1 INO80B-WBP1 KTN1-AS1 LINC00158 LINC00184 LINC00221 LINC00238 LINC00242 LINC00381 LINC00671 LINC00700 LOC100130476 LOC100132287 LOC100133920 LOC100271722 LOC100272228 LOC100289019 LOC100505474 LOC100505776 LOC100506035 LOC100506835 LOC100507410 LOC100507584 LOC284933 LOC340017 LOC728040 LOC728084 LOC728228 LOC729683 LOC729970 ORC2 PP2D1 SCARNA10 SLC8A1-AS1 SPANXA2-OT1 TCL1A TCL6 TM4SF19-TCTEX1D2 TPT1-AS1 TSNAX-DISC1 UBOX5-AS1
b
number of cells with cytokinetic bridges
0.08
0.04
0
****
****
****
linc00840
linc00899
C1QTNF1-AS1
loc729970
Ch-TOG ECT2 Cells Control GNG12-AS1 ASB16-AS1 ATP6V0E2-AS1 ATP6V1G2-DDX39B ATXN8OS CARD14 ELMO1 FAM182B FLJ36777 FLJ40852 FLJ42627 FOXN3-AS2 HPYR1 INO80B-WBP1 KTN1-AS1 LINC00158 LINC00184 LINC00221 LINC00238 LINC00242 LINC00381 LINC00671 LINC00700 LOC100130476 LOC100132287 LOC100133920 LOC100271722 LOC100272228 LOC100289019 LOC100505474 LOC100505776 LOC100506035 LOC100506835 LOC100507410 LOC100507584 LOC284933 LOC340017 LOC728040 LOC728084 LOC728228 LOC729683 LOC729970 ORC2 PP2D1 SCARNA10 SLC8A1-AS1 SPANXA2-OT1 TCL1A TCL6 TM4SF19-TCTEX1D2 TPT1-AS1 TSNAX-DISC1 UBOX5-AS1
c
% of multinucleated cells
50
30
10
8
4
0
****
****
linc00899
C1QTNF1-AS1
linc00840
loc729970
Ch-TOG ECT2 Cells Control GNG12-AS1 ASB16-AS1 ATP6V0E2-AS1 ATP6V1G2-DDX39B ATXN8OS CARD14 ELMO1 FAM182B FLJ36777 FLJ40852 FLJ42627 FOXN3-AS2 HPYR1 INO80B-WBP1 KTN1-AS1 LINC00158 LINC00184 LINC00221 LINC00238 LINC00242 LINC00381 LINC00671 LINC00700 LOC100130476 LOC100132287 LOC100133920 LOC100271722 LOC100272228 LOC100289019 LOC100505474 LOC100505776 LOC100506035 LOC100506835 LOC100507410 LOC100507584 LOC284933 LOC340017 LOC728040 LOC728084 LOC728228 LOC729683 LOC729970 ORC2 PP2D1 SCARNA10 SLC8A1-AS1 SPANXA2-OT1 TCL1A TCL6 TM4SF19-TCTEX1D2 TPT1-AS1 TSNAX-DISC1 UBOX5-AS1
d
% of viable cells
2000
1000
0
****
****
linc00899
C1QTNF1-AS1
linc00840
loc729970
Ch-TOG ECT2 Cells Control GNG12-AS1 ASB16-AS1 ATP6V0E2-AS1 ATP6V1G2-DDX39B ATXN8OS CARD14 ELMO1 FAM182B FLJ36777 FLJ40852 FLJ42627 FOXN3-AS2 HPYR1 INO80B-WBP1 KTN1-AS1 LINC00158 LINC00184 LINC00221 LINC00238 LINC00242 LINC00381 LINC00671 LINC00700 LOC100130476 LOC100132287 LOC100133920 LOC100271722 LOC100272228 LOC100289019 LOC100505474 LOC100505776 LOC100506035 LOC100506835 LOC100507410 LOC100507584 LOC284933 LOC340017 LOC728040 LOC728084 LOC728228 LOC729683 LOC729970 ORC2 PP2D1 SCARNA10 SLC8A1-AS1 SPANXA2-OT1 TCL1A TCL6 TM4SF19-TCTEX1D2 TPT1-AS1 TSNAX-DISC1 UBOX5-AS1

Supplementary Figure 2. Validation screen for lncRNAs involved in mitotic progression and cytokinesis.

Top 25 lncRNAs from screens A and B were selected and the validation screen was performed in HeLa cells stained with the same antibodies as used in screen B (PHH3, α-tubulin and γ-tubulin). Two biological replicates were performed with four technical replicates. The number of mitotic cells (mitotic index, ***a***), the number of cells with cytokinetic bridges (***b***), the number of multinucleated cells (***c***) and the number of viable cells (***d***) were calculated. *Ch-TOG* and *ECT2* siRNAs (both as red circles) were used as positive controls for mitotic index and multinucleation, respectively. Depletion of *ECT2* led to an increase in multinucleated cells, while depletion of *Ch-TOG* led to an increase in mitotic index. Cells treated with negative control siRNA (Control, Ambion) and cells alone are indicated as grey circles. Top lncRNA candidates involved in mitotic progression and cytokinesis are indicated as blue and orange circles, respectively. Statistical significance by two-tailed Student's t -test: **** P<0.0001. Source data are provided as a Source Data file.

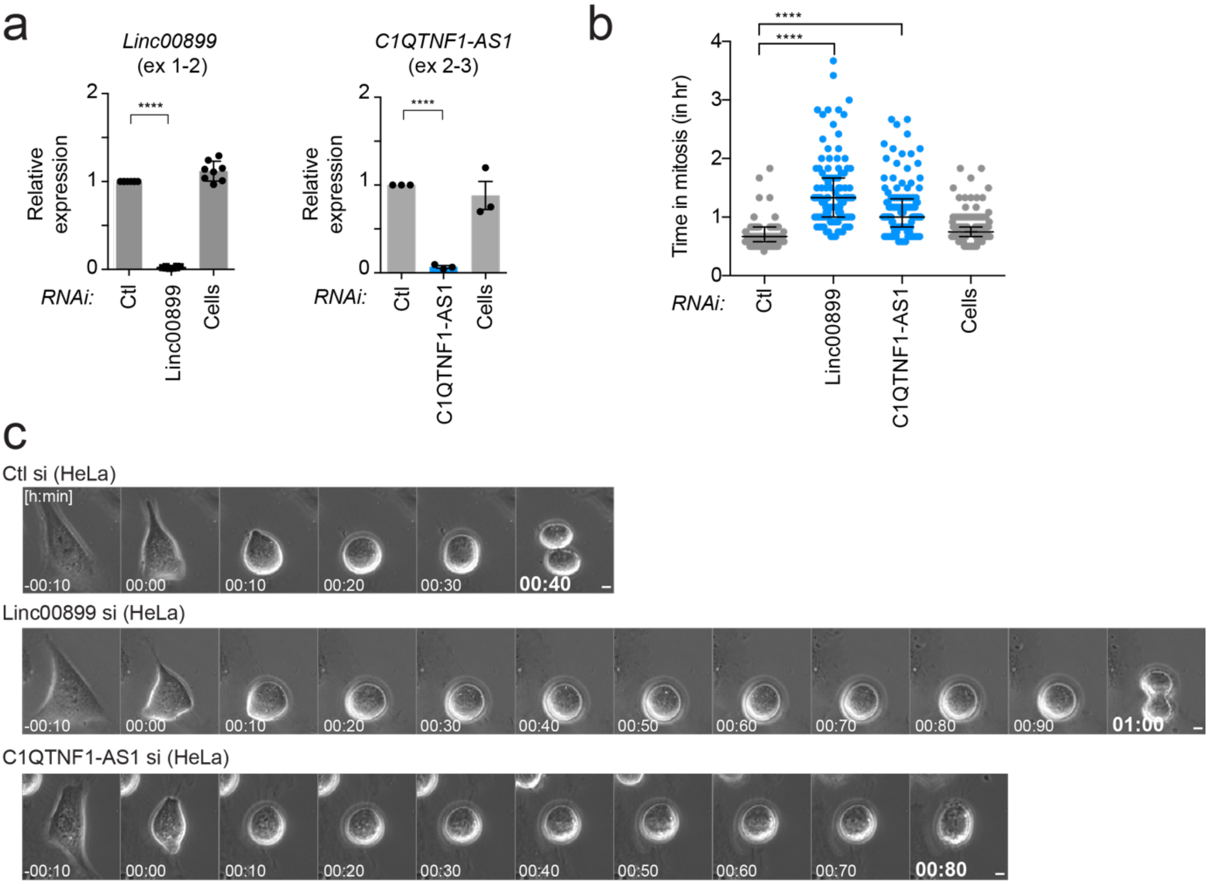

Supplementary Figure 3. RNAi-mediated depletion of *linc00899* and *C1QTNF1-AS1* leads to mitotic delay in HeLa cells.

a. Expression of *linc00899* and *C1QTNF1-AS1* after RNAi-mediated depletion of each lncRNA using pool of four siRNA sequences in HeLa cells, as quantified by qPCR. Results are also shown for cells treated with negative control siRNA (Ctl, from Ambion) and cells treated with transfection reagent alone (Cells). Expression values were compared to cells treated with negative control siRNA. Error bars, mean ± S.E.M. n = 3 - 8 biological replicates. Statistical significance by two-tailed Student's t -test: **** P<0.0001.

b. Quantification of mitotic progression by time-lapse microscopy imaging of HeLa cells after RNAi-mediated depletion of *linc00899* and *C1QTNF1-AS1*. Mitotic duration was defined from nuclear envelope breakdown (NEBD, t=0 mins) to anaphase onset using bright-field microscopy. Number of cells analysed is n=126 for negative control siRNAs (Ctl), n=118 for *linc00899* RNAi, n=129 for *C1QTNF1-AS1* RNAi and n=140 for cells treated with

transfection reagent alone (Cells). Bars show the median with interquartile range from 2 biological replicates. Statistical significance by Mann-Whitney test: ****P <0.0001.

c. Representative still images from time-lapse microscopy imaging show the mitotic delay in *linc00899* and *C1QTNF1-AS1*-depleted HeLa cells compared to cells treated with negative control siRNAs (Ctl). Scale bar, 5μm.

Source data are provided as a Source Data file.

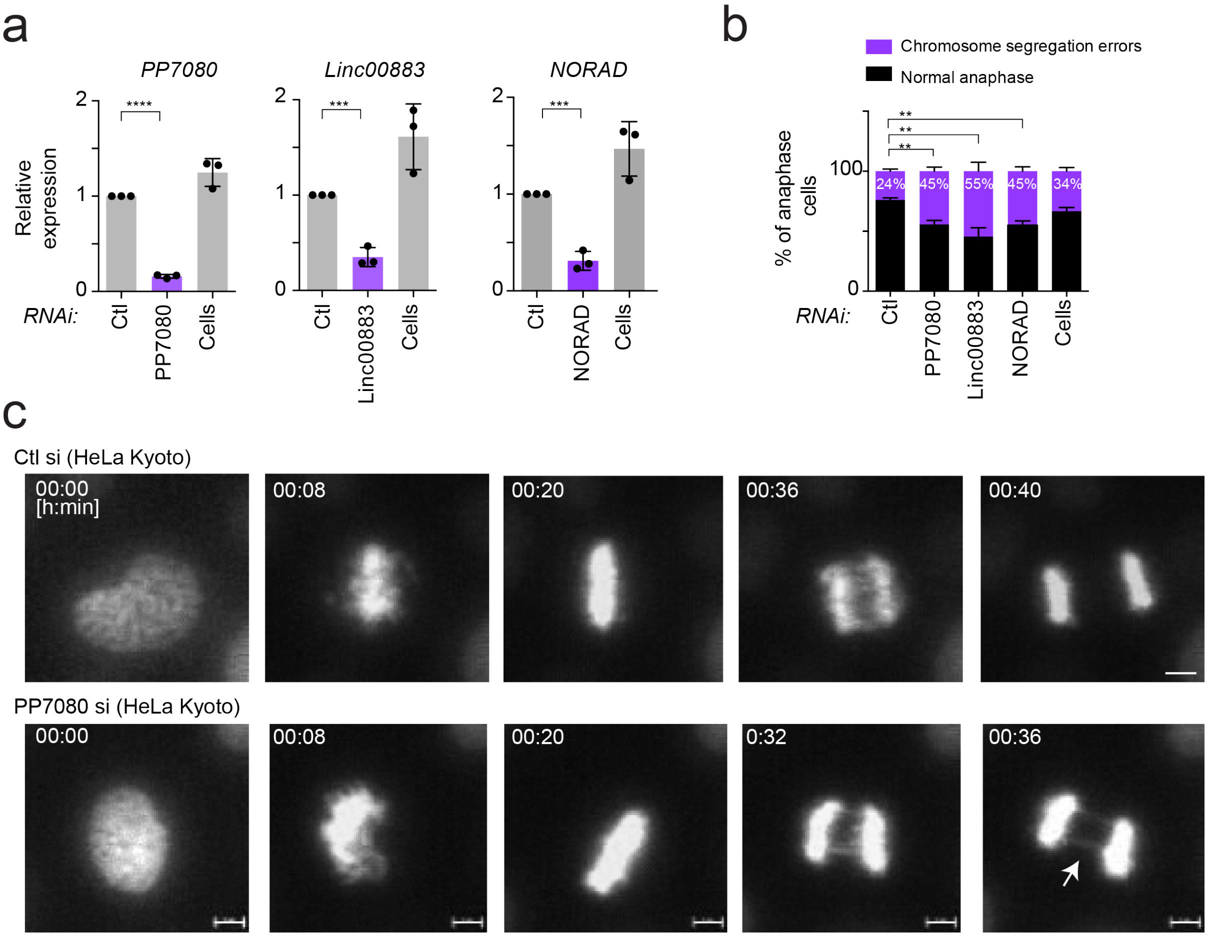

Supplementary Figure 4. RNAi-mediated depletion of *PP7080, linc00883* and *NORAD* leads to increased rate of chromosome segregation errors in HeLa cells.

a. Expression of *PP7080 (ENSG00000188242), linc00883 (ENSG00000243701) and NORAD* after RNAi-mediated depletion of each lncRNA using pool of four siRNA sequences in HeLa cells, as measured by qPCR. Results are also shown for cells treated with negative control siRNA (Ctl, from Ambion) and cells treated with transfection reagent alone (Cells). Expression levels were compared to the cells treated with control siRNA. n = 3 biological replicates. Statistical significance by two-tailed Student's t -test: *** P<0.001 and **** P<0.0001.

b. Quantification of chromosome segregation errors in HeLa Kyoto cells after depletion of each lncRNA with RNAi using time-lapse microscopy imaging. Number of anaphase cells analysed is n=110 for cells treated with transfection reagent alone (Cells), n=164 for negative control siRNAs (Ctl), n=140 for *NORAD* RNAi, n=140 for *PP7080* RNAi,

and n=171 for *linc00883* RNAi. Statistical significance by two-tailed Student's t-test: **P <0.01.

c. Representative still images of H2B-mCherry (white) from the time-lapse microscopy imaging in *PP7080*-depleted HeLa Kyoto cells expressing compared to cells treated with negative control siRNAs (Ctl). White arrow depicts chromosome segregation errors (chromatin bridges). Scale bar, 5µm.

Data are shown as mean ± S.E.M for (***a***) and (***b***). Source data are provided as a Source Data file.

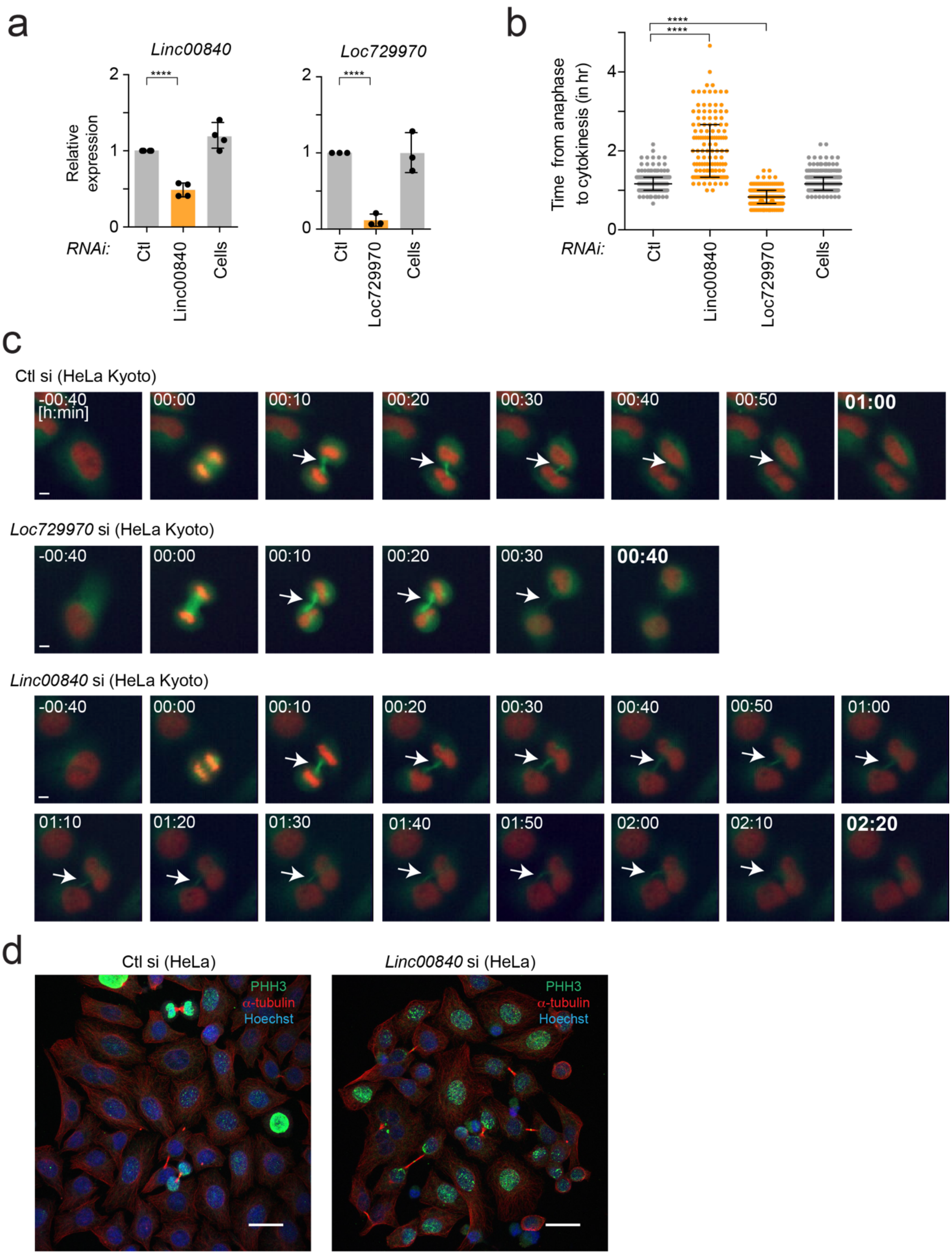

Supplementary Figure 5. RNAi-mediated depletion of *linc00840* and *loc729970* leads to cytokinesis defects in HeLa cells.

a. Expression of *linc00840 (ENSG00000226808)* and *loc729970 (ENSG00000235501)* after RNAi-mediated depletion of each lncRNA using a pool of four siRNA sequences in HeLa cells, as measured by qPCR. Results are also shown for cells treated with negative control siRNAs (Ctl, from Ambion) or cells treated with transfection reagent alone (Cells). Error bars, S.E.M. n = 3 - 4 biological replicates. Statistical significance by two-tailed Student's t -test: **** P<0.0001.

b. Quantification of cytokinesis defects in HeLa Kyoto cells expressing eGFP α-tubulin (green) and H2B-mCherry (red) and progressing through mitosis after depletion of each lncRNA with RNAi, as measured by time-lapse microscopy imaging. The time in cytokinesis was measured from anaphase onset (t=0 mins) to completion of abscission. The number of cells analysed was n=160 for cells treated with negative control siRNAs (Ctl), n=187 for cells treated with transfection reagent alone (Cells), n=130 for *linc00840* RNAi and n=238 for *loc729970* RNAi. Bars show the median with interquartile range from 3 biological replicates. Statistical significance by Mann-Whitney test: ****P <0.0001.

c. Representative still images from the time-lapse microscopy in *linc00840* and *loc729970*-depleted HeLa Kyoto cells showing the time required to cleave the cytokinetic bridge, compared to cells treated with negative control siRNAs (Ctl). White arrow depicts cytokinetic bridge. Scale bar, 5µm.

d. Representative confocal images of control and *linc00840*-depleted HeLa cells stained with α-tubulin, PHH3 and Hoechst (DNA). Scale bar, 20µm. Note increased number of cytokinetic bridges in *linc00840*-depleted HeLa cells.

Source data are provided as a Source Data file.

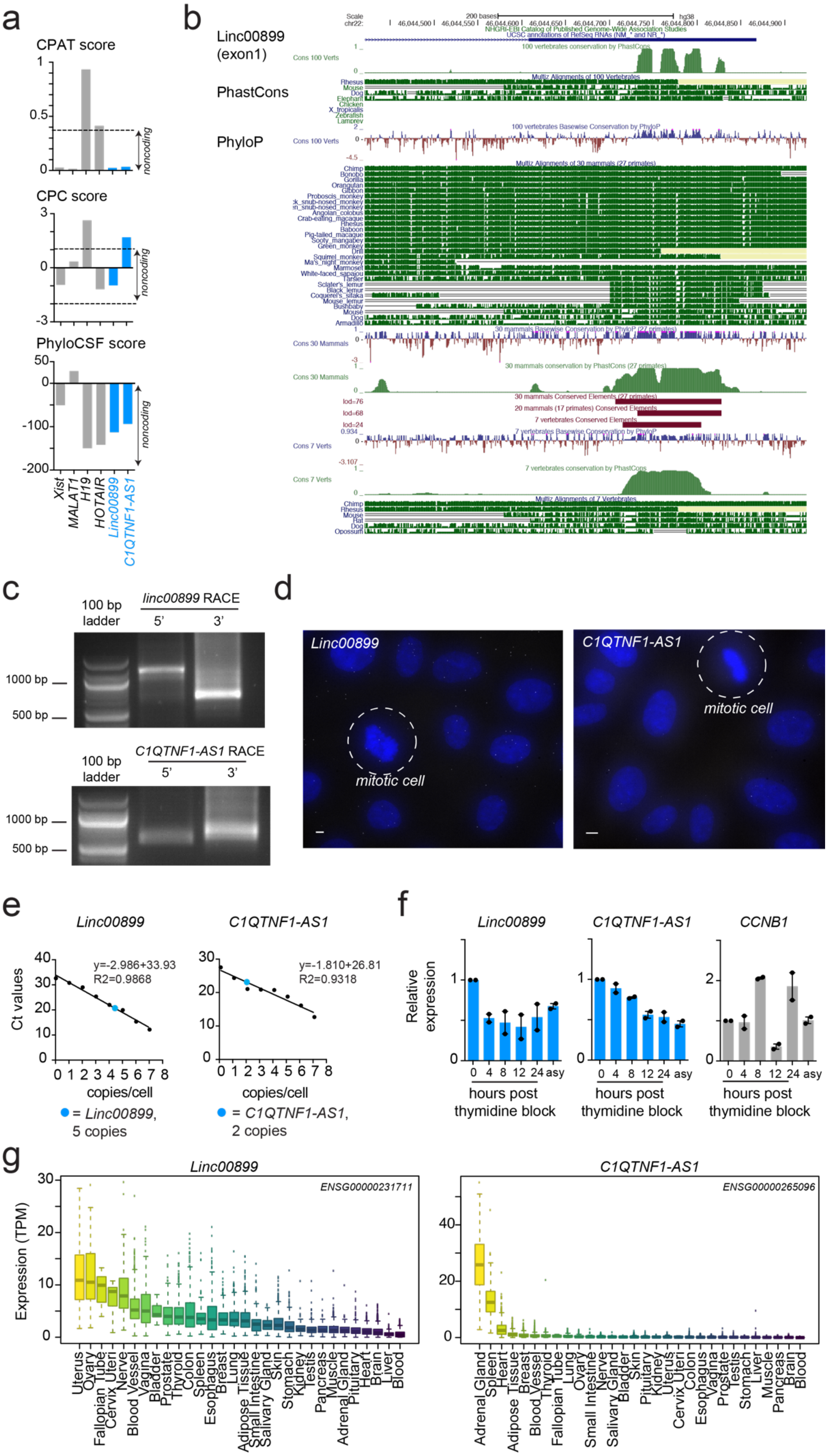
a
CPAT score
CPC score
PhyloCSF score
noncoding
Xist
MALAT1
H19
HOTAIR
Linc00899
C1QTNF1-AS1
b
Linc00899 (exon1)
PhastCons
PhyloP
c
100 bp ladder
linc00899 RACE
5'
3'
1000 bp
500 bp
C1QTNF1-AS1 RACE
d
Linc00899
C1QTNF1-AS1
mitotic cell
e
Linc00899
C1QTNF1-AS1
Ct values
y=-2.986+33.93
R2=0.9868
y=-1.810+26.81
R2=0.9318
copies/cell
= Linc00899, 5 copies
= C1QTNF1-AS1, 2 copies
f
Linc00899
C1QTNF1-AS1
CCNB1
Relative expression
hours post thymidine block
asy
g
Linc00899
C1QTNF1-AS1
ENSG00000231711
ENSG00000265096
Expression (TPM)

Supplementary Figure 6. Molecular and cellular characteristics of *linc00899* and *C1QTNF1-AS1.*

a. Computational analysis of the *linc00899* and *C1QTNF1-AS1* sequences using a variety of protein coding potential tools. Low protein-coding potentials were detected by CPAT, CPC and PhyloCSF score. For comparison, scores are also shown for a number of well-known lncRNAs as positive controls (black text).

b. Sequence conservation of exon 1 of *linc00899,* as scored with PhastCons and PhyloP. Results are shown as tracks on the UCSC genome browser.

c. Identification of the 5' and 3' ends of *linc00899* and *C1QTNF1-AS1* with RACE in HeLa cells. Isoforms were identified with lengths ranging from 1144 to 1562 bp for *linc00899* and 864 to 952 bp for *C1QTNF1-AS1*.

d. Single molecule RNA FISH of *linc00899* (left) and *C1QTNF1-AS1* (right) in interphase and mitotic cells (circle) using exonic probes against each of the mature transcript. Representative images are shown from at least 3 biological replicates. DNA is stained with DAPI and shown in blue, while probes are shown in white. Scale bar represents 20µm.

e. Copy numbers of *linc00899* and *C1QTNF1-AS1* in HeLa cells, determined by qPCR. RNA was extracted from known number of HeLa cells, and the copy number per cell was computed using the standard curve method for Ct values against dilutions of a lncRNA DNA template of known concentration. *Linc00899* is present at five copies while *C1QTNF1-AS1* at two copies per cell. n = 5 biological replicates.

f. Expression levels of *linc00899* and *C1QTNF1-AS1* in synchronized HeLa cells using double thymidine block as measured by qPCR. *Cyclin B1 (CCNB1)* was used as a positive control to mark cells in G2/M phase of the cell cycle. Expression levels were standardised to time point 0 hr. Results are also shown for asynchronous cells (asy). Error bars, S.E.M. n = 2 biological replicates.

g. Expression values for *linc00899* and *C1QTNF1-AS1* were obtained from version 7 of the GTEx RNA-seq expression dataset (*https://gtexportal.org/home/datasets*) for normal

tissues and are shown as boxplots of transcripts per million (TPM). Tissues are sorted in order of decreasing median expression across all GTEx samples for each lncRNA. Points for each tissue represent samples that lie more than 1.5-fold interquartile ranges from the third quartile.

Source data are provided as a Source Data file.

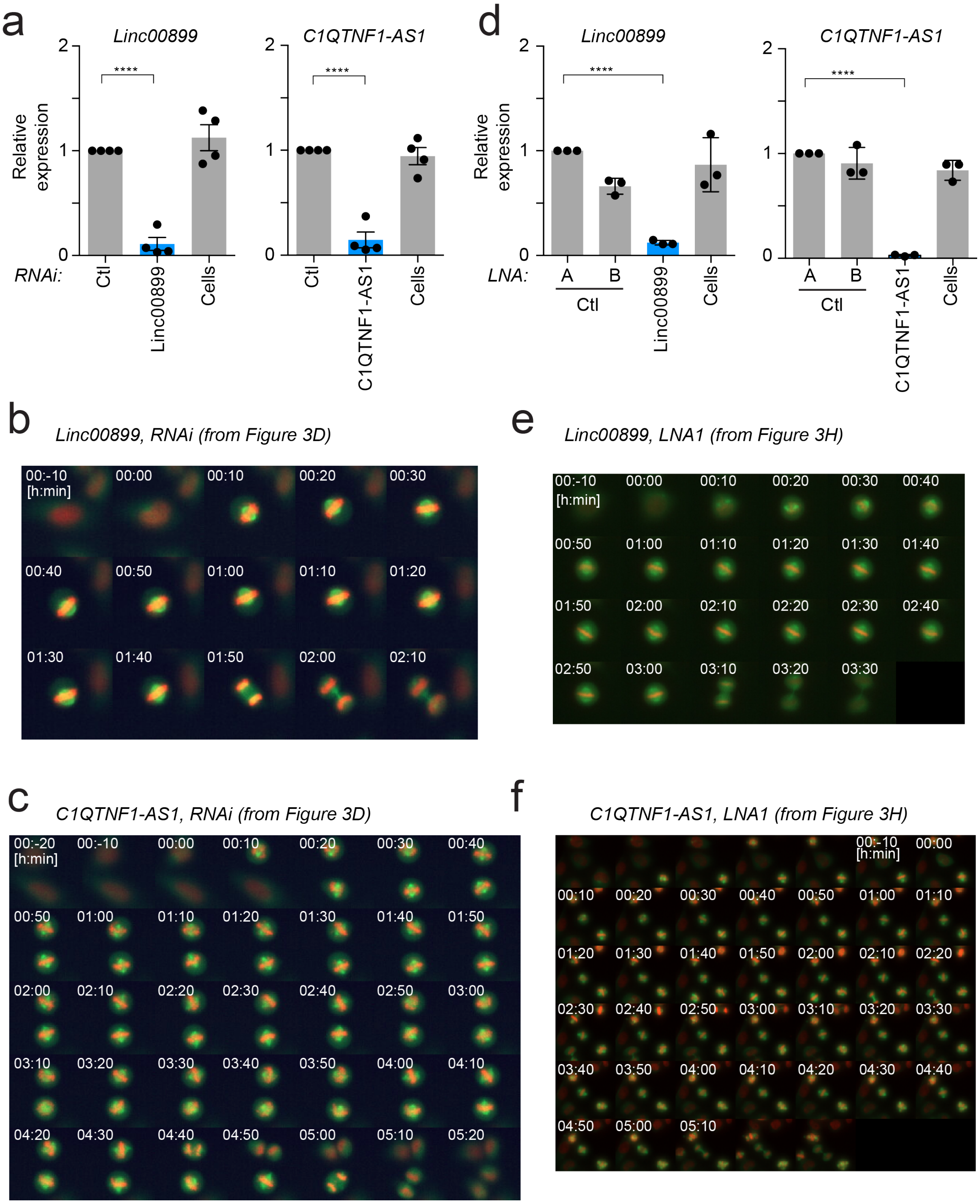

Supplementary Figure 7. RNAi- and LNA-mediated depletion of *linc00899* and *C1QTNF1-AS1* in HeLa Kyoto cells.

a. Expression of *linc00899 and C1QTNF1-AS1* in HeLa Kyoto cells after their depletion with RNAi using a pool of four siRNA sequences, as measured with qPCR. Results are also shown for cells treated with negative control siRNAs (Ctl, from Ambion) or cells

treated with transfection reagent alone (Cells). n = 4 biological replicates. Statistical significance by two-tailed Student’s t -test: **** P<0.0001.

b. One representative series of still images from time-lapse microscopy in *linc00899*-depleted HeLa Kyoto cells.

c. One representative series of images from time-lapse microscopy in *C1QTNF1-AS1*-depleted HeLa Kyoto cells.

d. Expression of *linc00899* and *C1QTNF1-AS1* in HeLa Kyoto cells after their depletion with LNA1 gapmers, as measured with qPCR. Results are also shown for cells treated with negative control LNA A and B (Ctl A and B) or with transfection reagent alone (Cells). n = 3 biological replicates. Statistical significance by two-tailed Student’s t -test: **** P<0.0001.

e. One representative series of still images from time-lapse microscopy in *linc00899*-depleted HeLa Kyoto cells.

f. One representative series of images from time-lapse microscopy in *C1QTNF1-AS1*-depleted HeLa Kyoto cells.

Data are shown as mean ± S.E.M for (***a***) and (***d***). The cropped series are shown in Figure 3. Source data are provided as a Source Data file.

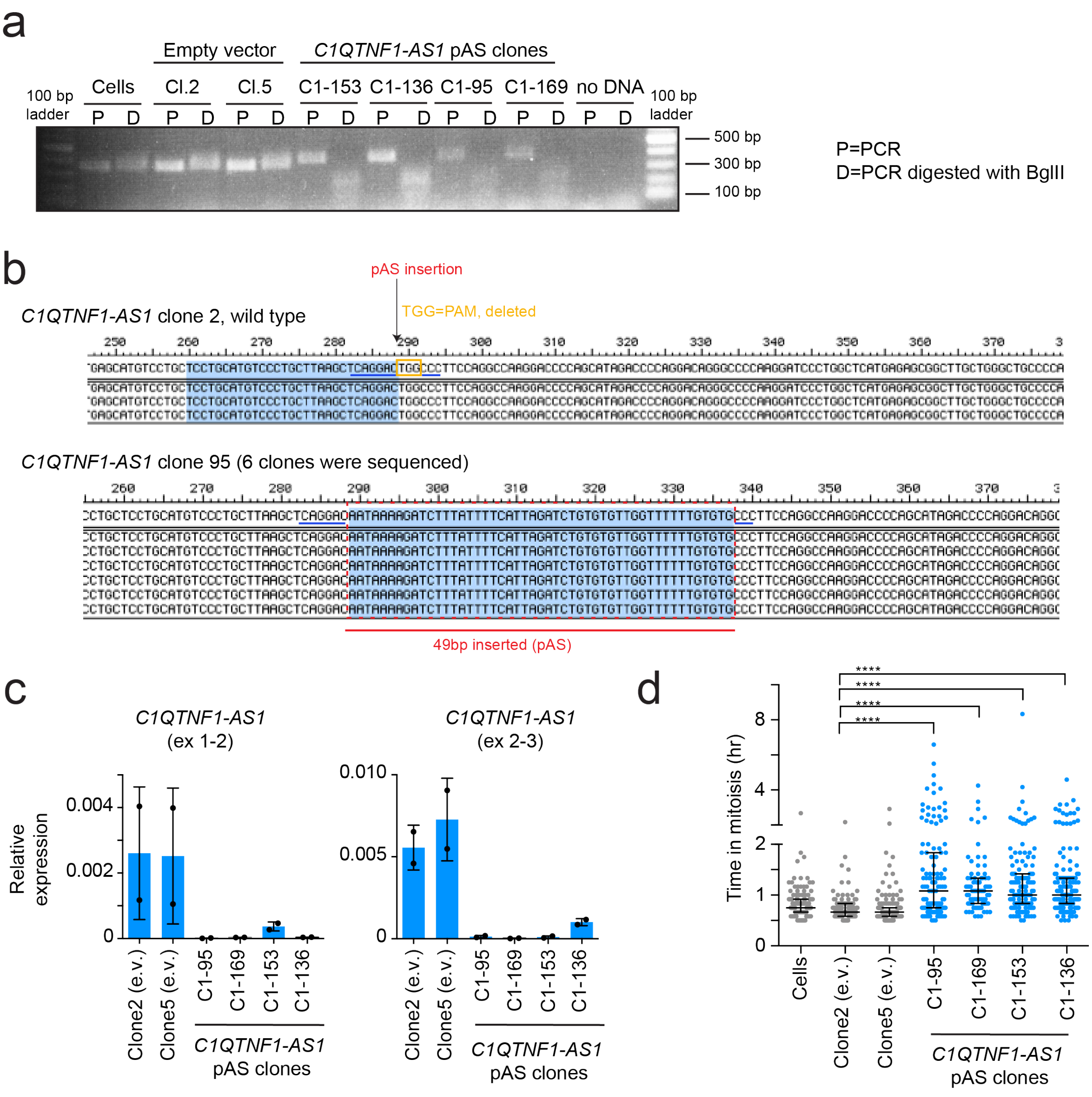

Supplementary Figure 8. Generation of CRISPR clones with insertion of poly(A) signal to terminate transcription of *C1QTNF1-AS1.*

a. Detection of genomic DNA containing poly(A) sequence (pAS) insertion at the *C1QTNF1*-AS1 locus, as determined by PCR. Results are shown for HeLa cells alone (Cells), clones stably integrated with an empty vector PX458 (e.v., clones 2 and 5, negative controls), and four different *C1QTNF1-AS1* clones transfected with the pAS-*C1QTNF1-AS1* insert (clones C1-95, C1-169, C1-153 and C1-136). After PCR, a small aliquot was digested with *Bgl*II. In cells with no insert, the PCR product should be 358 bp and should not be affected by digestion. If pAS (49 bp) was inserted, the PCR product

should be 404 bp (49 bp+358 bp) before digestion, and should yield two products of 149 bp and 237 bp after digestion. The 100 bp ladder is shown on each side of the 2% agarose gel. PCR primers are provided in the Supplementary Table 8.

b. Representative sequences after pAS insertion into the *C1QTNF1-AS1* sequence from clone 95, compared to negative control clone 2. Genomic DNA was extracted from the cells and cloned into pJET BLUNT. After transformation, several bacteria clones were subjected to Sanger sequencing using pJET Forward primers.

c. Expression of *C1QTNF1-AS1* after insertion of pAS into exon 1 of the *C1QTNF1-AS1* locus, as quantified by qPCR. The pAS insertion allows transcription at the *C1QTNF1-AS1* locus while inhibiting production of the *C1QTNF1-AS1* transcript. Results are shown for cells transfected with the empty vector PX458 (e.v., clones 2 and 5 as negative controls) and four *C1QTNF1-AS1* pAS clones. qPCR primers against exons 1-2 and 2-3 were used to quantify the knockdown efficiency of *C1QTNF1-AS1*. Error bars, S.E.M. n = 2 biological replicates.

d. Quantification of mitotic progression by time-lapse microscopy imaging in four HeLa *C1QTNF1-AS1* pAS clones. Mitotic duration was measured from nuclear envelope breakdown (NEBD, t=0 mins) to anaphase onset using the bright-field microscopy. The number of mitotic cells analysed was n=137 for HeLa cells alone (Cells), n=109 for the negative control clone 2, n=145 for negative control clone 5, n=121 for *C1QTNF1-AS1* pAS clone 95, n=84 for pAS clone 169, n=131 for pAS clone 153, and n=156 for pAS clone 136. Bars show median with interquartile range from two biological replicates. Statistical significance by Mann-Whitney test: ****P <0.0001.

Source data are provided as a Source Data file.

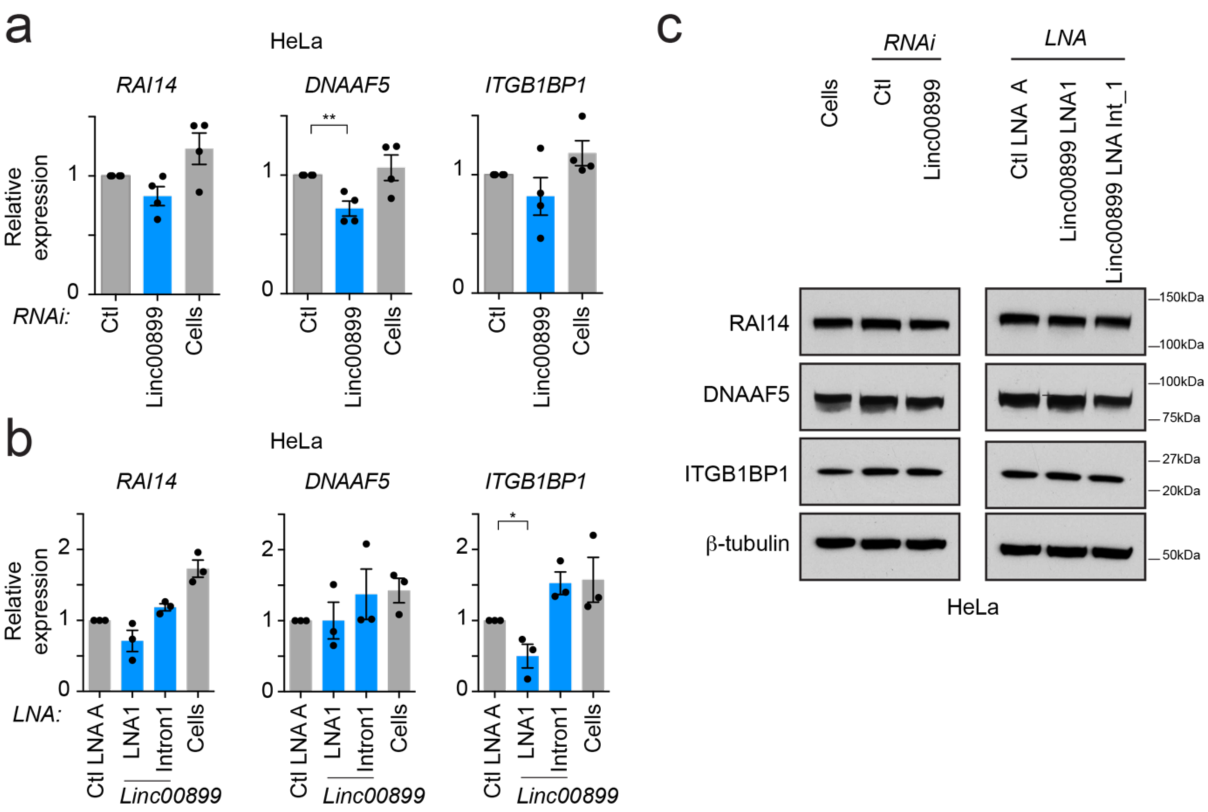

Supplementary Figure 9. Expression of *RAI14*, *DNAAF5* and *ITGB1BP1* after depletion of *linc00899* using RNAi and LNA gapmers in HeLa cells.

a-b. Expression of *RAI14, DNAAF5* and *ITGB1BP1* after depletion of *linc00899* using RNAi (***a***) and LNA gapmers (***b***; LNA gapmer 1 and LNA against the intron 1). Results are also shown for cells treated with negative control siRNA (Ctl, from Ambion) or negative control LNA (Ctl LNA A), and for cells treated with transfection reagent alone (Cells). Expression values are shown relative to cells treated with negative controls. Error bars, S.E.M. n = 3 - 4 biological replicates. Statistical significance by two-tailed Student's *t*-test: * P<0.1 and ** P<0.01.

c. Western blot of *RAI14, DNAAF5* and *ITGB1BP1* levels after depletion of *linc00899* in HeLa cells, as measured in (***a***) and (***b***). β-tubulin was used as a loading control.

Source data are provided as a Source Data file.

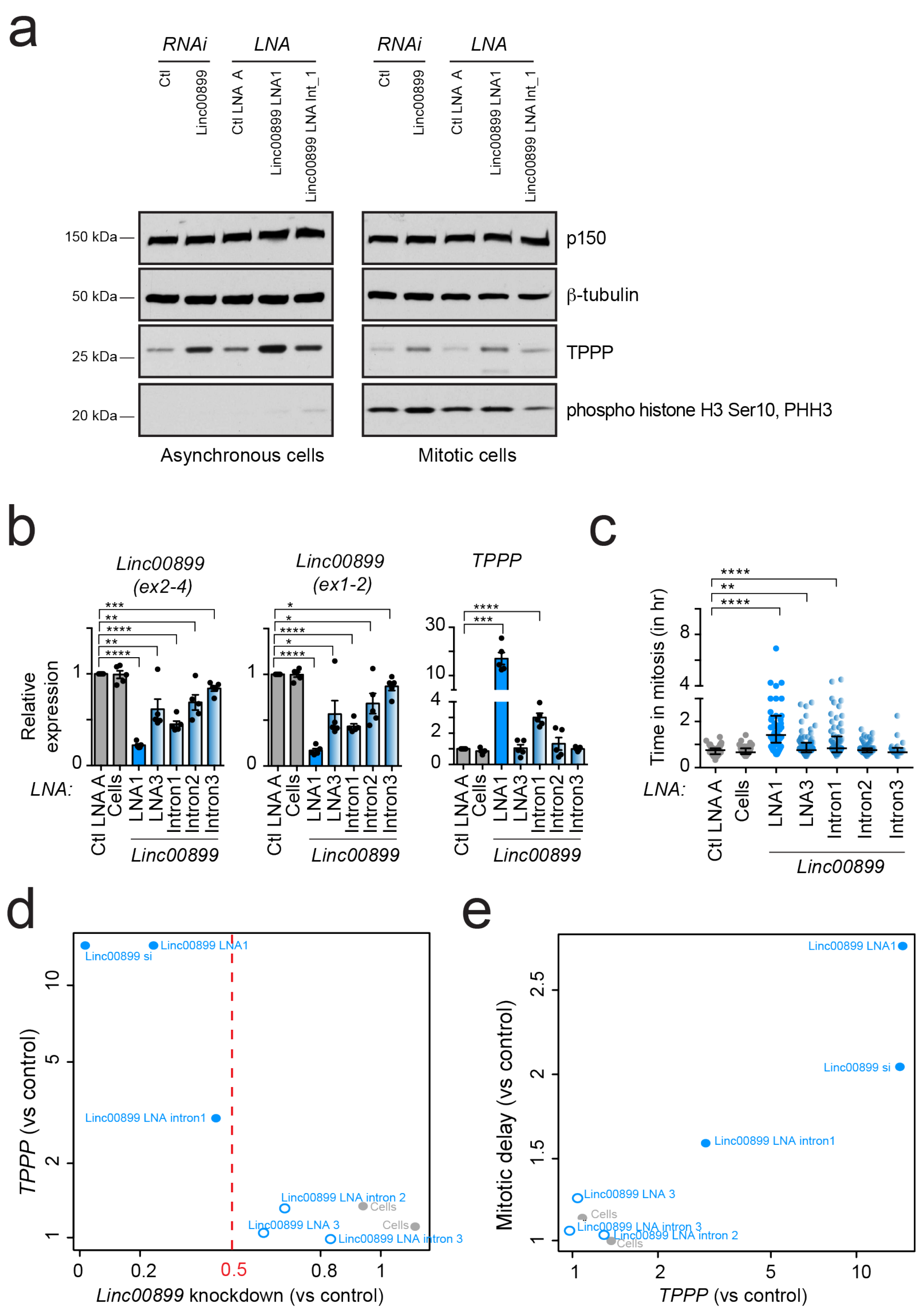

Supplementary Figure 10. Depletion of *linc00899* using additional LNA gapmers targeting either exon 1 (LNA 3) or intronic regions of *linc00899* (intronic LNAs).

a. Western blot of TPPP levels after depletion of *linc00899* in asynchronous (left) and mitotic cells (right) compared to negative control siRNA (Ctl, from Ambion) or negative control LNA (Ctl LNA A). β-tubulin and p150 were used as two loading controls. Mitotic

cell extracts were obtained by mitotic shake-off of monastrol-treated cells and PHH3 antibody was used as a control to show enrichment of mitotic cells.

b. Expression of *linc00899* and *TPPP* after depletion of *linc00899* using LNA gapmer 1 (as in Figure 3) with additional LNA gapmers targeting either different region of exon 1 (LNA 3) or different intronic regions (intron 1-3). Results are also shown for cells treated with negative control LNA A (Ctl LNA A) and for cells treated with transfection reagent alone (Cells). Expression values are shown relative to cells treated with negative control LNA A. Error bars, S.E.M. n = 5 biological replicates. Statistical significance by two-tailed Student's *t*-test: * P<0.1, ** P<0.01, *** P<0.001 and **** P<0.0001.

c. Quantification of mitotic progression in HeLa cells by time-lapse microscopy imaging after LNA-mediated depletion of *linc00899* using the additional LNA gapmers as in (***b***). Mitotic duration was measured from NEBD (t=0 mins) to anaphase onset by bright-field microscopy. Number of mitotic cells analyzed was n= 96 for negative control LNA A (Ctl LNA A), n=50 for cells treated with transfection reagent alone (Cells), n=53 for *linc00899* LNA 1, n=82 for *linc00899* LNA 3, n=74 for *linc00899* LNA targeting intron 1, n=78 for *linc00899* LNA targeting intron 2, and n=22 for *linc00899* LNA targeting intron 3. Bars show median with interquartile range from 2 biological replicates. Statistical significance by Mann-Whitney test: ***P*<0.01 and *****P*<0.0001.

d. Upregulation of *TPPP* expression with respect to *linc00899* knockdown efficiency, after depleting *linc00899* with a variety of LOF methods. Expression was quantified using qPCR and calculated relative to the appropriate negative control for each method. Each value represents the average of 4-5 replicates per condition. Results are also shown for cells treated with transfection reagent alone (Cells, in grey). LOF methods achieving more than 50% depletion are denoted with the blue closed circles.

e. Fold increase in the mitotic delay upon depletion of *linc00899* with a variety of LOF methods, with respect to *TPPP* upregulation. All values were computed relative to the appropriate negative control for each LOF method and represent the average of 4-5 replicates per condition. Points are denoted as described in (***b***).

Source data are provided as a Source Data file.

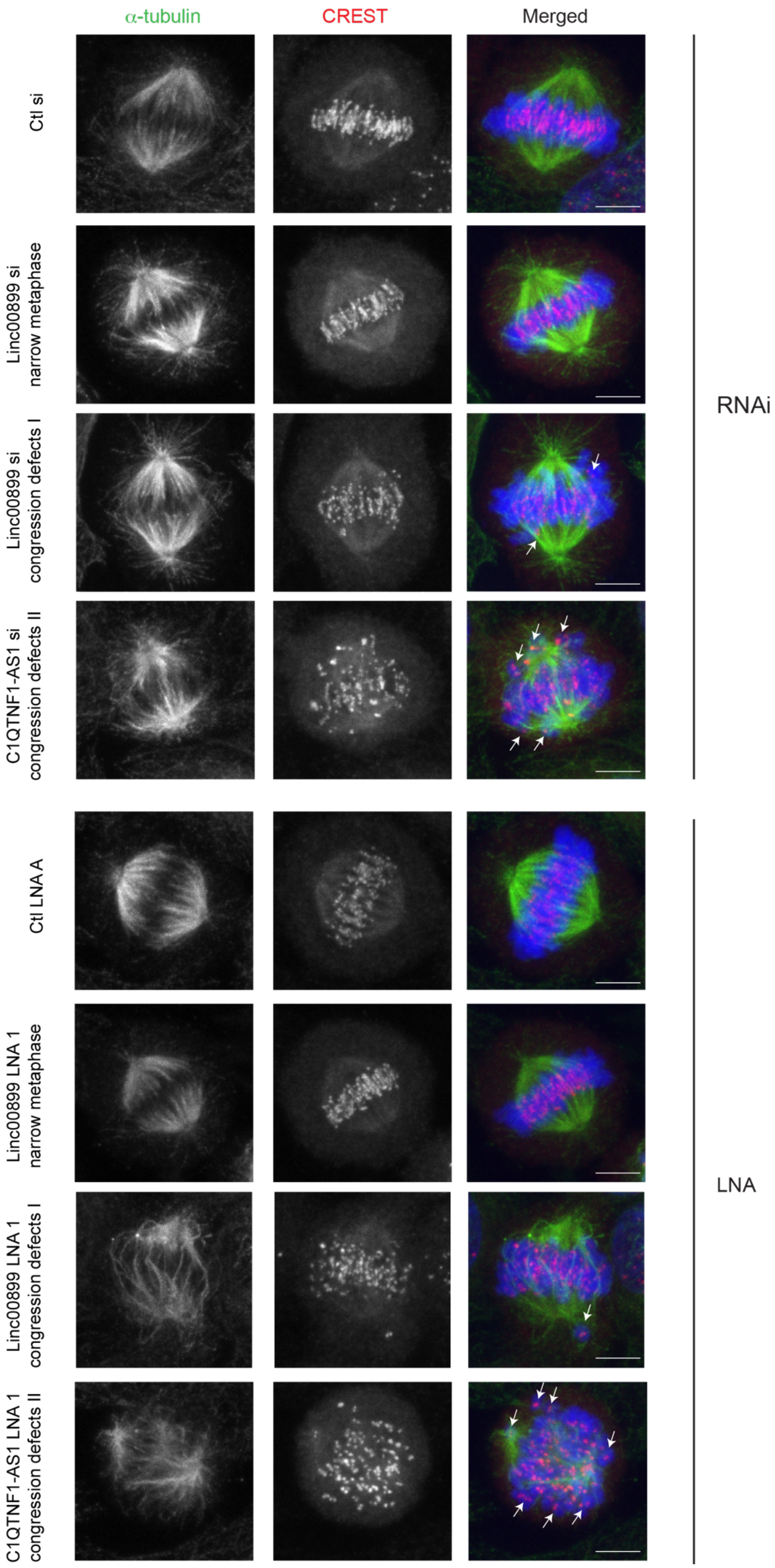
α-tubulin
CREST
Merged
Ctl si
Linc00899 si
narrow metaphase
Linc00899 si
congression defects I
C1QTNF1-AS1 si
congression defects II
RNAi
Ctl LNA A
Linc00899 LNA 1
narrow metaphase
Linc00899 LNA 1
congression defects I
C1QTNF1-AS1 LNA 1
congression defects II
LNA

Supplementary Figure 11. Representative confocal images of *linc00899* and *C1QTNF1-AS1*-depleted HeLa cells using RNAi and LNA gapmers.

The cells were stained with α-tubulin (microtubules, green), CREST (kinetochores, red) and Hoechst (DNA, blue). Scale bar, 5μm.

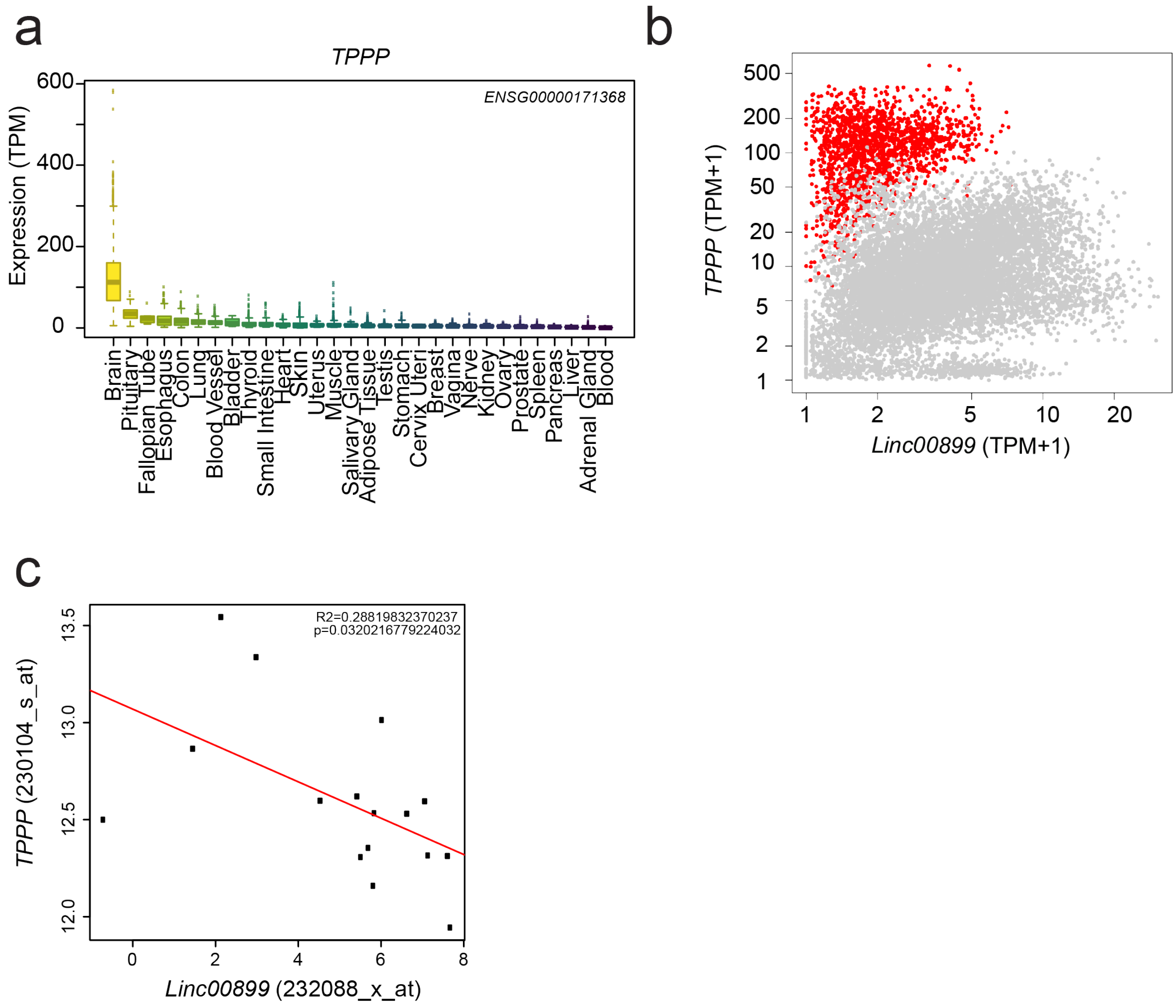

Supplementary Figure 12. Expression of *TPPP* and *linc00899* in humans.

a. Expression values for *TPPP* were obtained from version 7 of the GTEx RNA-seq expression dataset (*https://gtexportal.org/home/datasets*) for normal tissues and are shown as boxplots of transcripts per million (TPM). Tissues are sorted by decreasing median expression.

b. Expression of *TPPP* relative to *linc00899* across all samples in the GTEx dataset. Each point represents a single sample, with all brain samples coloured in red.

c. Expression of *TPPP* relative to *linc00899* across all samples in the GSE52139 dataset. Each point corresponds to one multiple sclerosis patient, and all gene expression values represent log_2-transformed probe intensities. The line of best fit is shown in red, with an R^2 value of 0.29 and a p-value of 0.03.

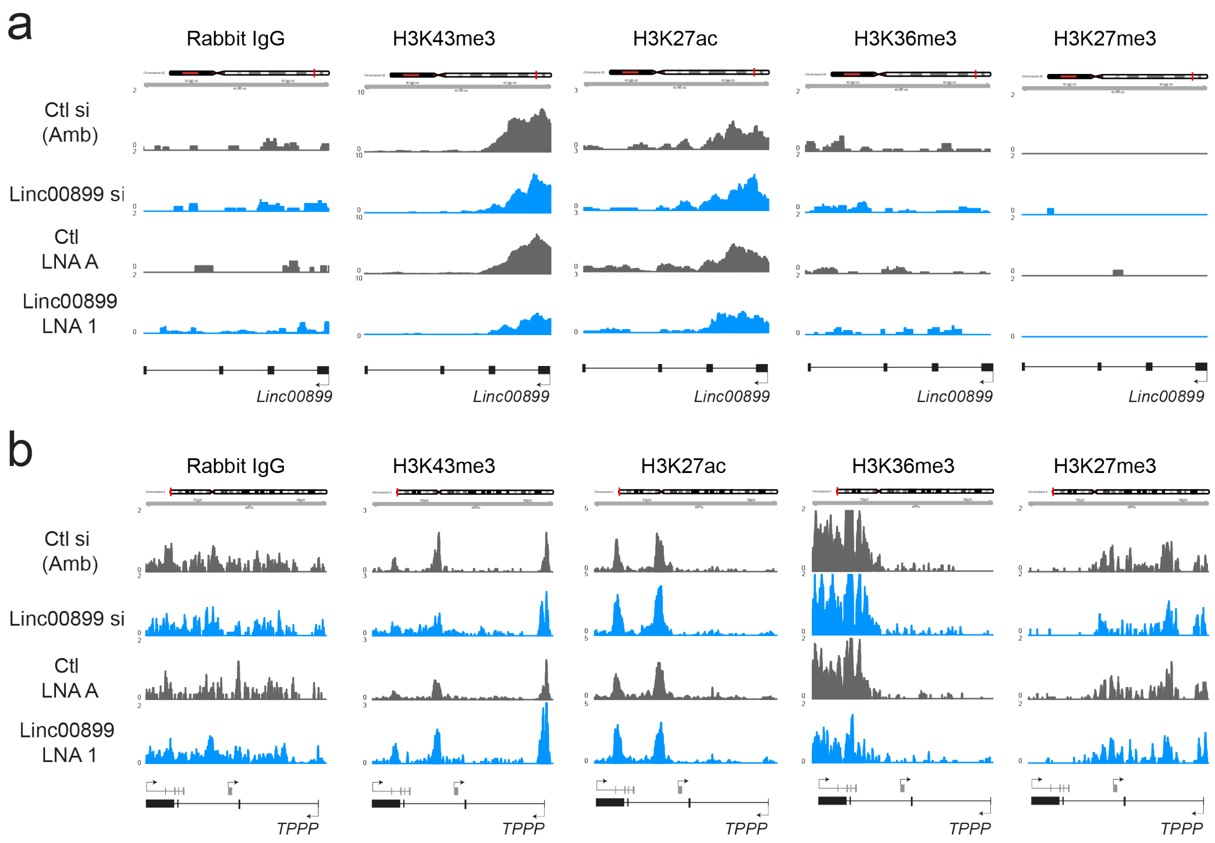

Supplementary Figure 13. Chromatin landscape at the *linc00899* and *TPPP* locus after depletion of *linc00899*.

CUT&RUN profiling of active (H3K4me3, H3K27ac, H3K36me3) and repressive (H3K27me3) histone modifications at the *linc00899* (***a***) and *TPPP* (***b***) locus after depletion of *linc00899* with RNAi and LNAs in HeLa cells. Coverage tracks are also shown for negative control siRNAs (Ctl, from Ambion) and negative control LNA A (Ctl LNA A). Each track represents the average normalized count-per-million at each base position across two biological replicates.

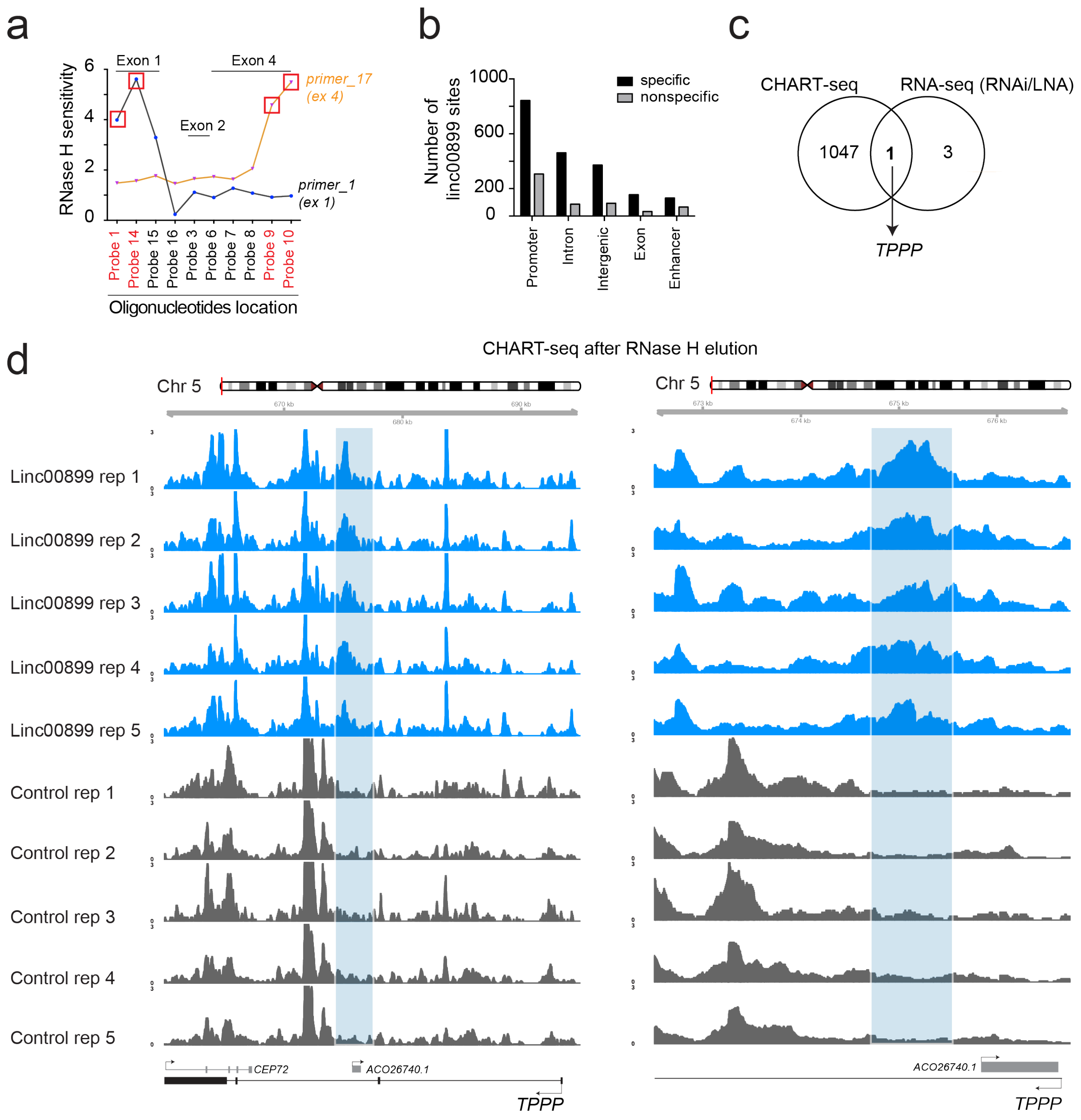

Supplementary Figure 14. Statistics for *linc00899* CHART-seq.

a. Mapping oligonucleotide binding to the *linc00899* transcript by RNase H sensitivity assays. Several antisense oligonucleotides were designed that directly hybridized to endogenous *linc00899* and RNase H mapping was used to identify oligonucleotides binding to accessible regions of the transcript. A cocktail of four such oligonucleotides were used to purify chromatin complexes containing *linc00899* transcript, whereas sense DNA oligonucleotides were used as controls to account for any non-specific binding of the probes to the DNA locus. Each DNA oligonucleotide was added to cross-

linked, sheared chromatin from HeLa cells to form RNA-DNA hybrids with the lncRNA transcript. Samples were treated with RNase H to digest the RNA in the RNA-DNA hybrids and incubated with DNase I to remove genomic DNA. RT-qPCR was performed to quantify RNase H cleavage of *linc00899*, using primers targeting exon 1 (primer 1) or exon 4 (primer 17) of *linc00899* (Supplementary Table 6). RNase H sensitivity was calculated based on the ratio of cleaved to uncleaved transcript, using no oligonucleotide as a control reaction. Different oligonucleotides spanning the *linc00899* transcript were tested, and the cocktail mix of probe 1 (exon 1), probe 14 (exon 1), probe 9 (exon 4) and probe 10 (exon 4) was used for CHART-seq.

b. Number of *linc00899* binding sites in different genomic contexts at an empirical FDR of 30%. We identified ~1964 locations with significant increases in coverage upon antisense pulldown compared to sense control. These putative *linc00899* binding sites were mostly distributed within promoters and introns. Specific binding sites were defined as those with significantly increased coverage in the antisense pulldown, while non-specific sites were defined as those with increased coverage in the sense pulldown (i.e., the negative control).

c. Overlap between the set of genes (1048) bound by *linc00899* in CHART-seq and the set of genes that were differentially expressed in the same direction in RNA-seq after LNA or RNAi-mediated depletion (see Fig. 4b). The only gene in the intersection was *TPPP*.

d. CHART-seq coverage tracks at the *TPPP* locus after pulldown with antisense (blue) or sense oligonucleotides (grey). Coverage is shown for 5 biological replicates (left), with a zoomed-in view of the site with the most significant change in coverage (right).

Source data are provided as a Source Data file.

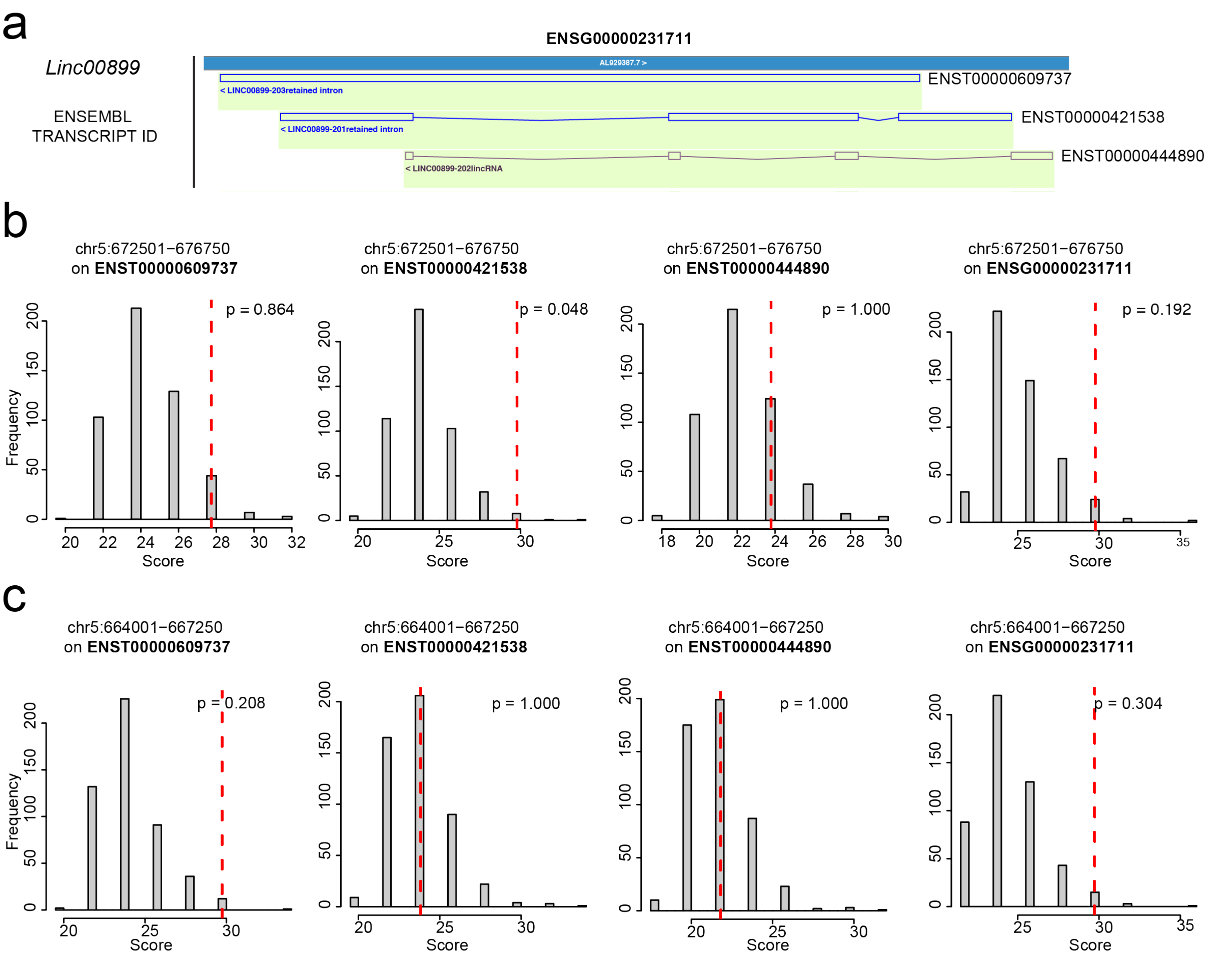

Supplementary Figure 15. Complementarity between the *TPPP* genomic sequence and the transcript sequence of *linc00899.*

Smith-Waterman local alignment scores between the *linc00899* transcript and the sequences of the genomic intervals within *TPPP* bound by *linc00899* (as detected by CHART-seq). We examined three isoforms of *linc00899* from Ensembl annotation along with the premature transcript (***a***). For each interval (***b, c***), we performed a local alignment between each *linc00899* sequence to the genome sequence, taking the maximum score across both strands of the latter. We repeated this process after shuffling the sequence to obtain a null distribution of 100 alignment scores. Genuine matches should exhibit a real alignment score (red) that is much greater than the random distribution (grey).
