# Supplementary material for: A high-content RNAi screen reveals multiple roles for long noncoding RNAs in cell division
Source: Nat Commun. 2020 Apr 15;11:1851. doi: 10.1038/s41467-020-14978-7 (PMC7160116; doi:10.1038/s41467-020-14978-7)
Supplement: Supplementary file 3 — Reporting Summary [file 41467_2020_14978_MOESM3_ESM.pdf]

nature research

Corresponding author(s): Lovorka Stojic, Fanni Gergely and Duncan Odom; NCOMMS-19-16524B

Last updated by author(s): 24.12.2019

Reporting Summary

Nature Research wishes to improve the reproducibility of the work that we publish. This form provides structure for consistency and transparency in reporting. For further information on Nature Research policies, seeAuthors & Referees and theEditorial Policy Checklist .

Statistics

For all statistical analyses, confirm that the following items are present in the figure legend, table legend, main text, or Methods section.

n/a	Confirmed	
☐	☒	The exact sample size (*n*) for each experimental group/condition, given as a discrete number and unit of measurement
☐	☒	A statement on whether measurements were taken from distinct samples or whether the same sample was measured repeatedly
☐	☒	The statistical test(s) used AND whether they are one- or two-sided<br>*Only common tests should be described solely by name; describe more complex techniques in the Methods section.*
☐	☒	A description of all covariates tested
☐	☒	A description of any assumptions or corrections, such as tests of normality and adjustment for multiple comparisons
☐	☒	A full description of the statistical parameters including central tendency (e.g. means) or other basic estimates (e.g. regression coefficient) AND variation (e.g. standard deviation) or associated estimates of uncertainty (e.g. confidence intervals)
☐	☒	For null hypothesis testing, the test statistic (e.g. *F*, *t*, *r*) with confidence intervals, effect sizes, degrees of freedom and *P* value noted<br>*Give P values as exact values whenever suitable.*
☒	☐	For Bayesian analysis, information on the choice of priors and Markov chain Monte Carlo settings
☒	☐	For hierarchical and complex designs, identification of the appropriate level for tests and full reporting of outcomes
☒	☐	Estimates of effect sizes (e.g. Cohen's *d*, Pearson's *r*), indicating how they were calculated

Our web collection on statistics for biologists contains articles on many of the points above.

Software and code

Policy information about availability of computer code

Data collection	qPCR data were collected using the Applied Biosystems QuantStudio™ Real-Time PCR Software.<br>Western blot data were collected using the Colenta MEDIPHOT 900E X-ray film processor.
Data analysis	We used the R programming language (version 3.5.1) with the edgeR (version 3.22.0), limma (version 3.36.0) and csaw packages (version 1.14.0) from the open-source Bioconductor project. For read alignment, we used subread 1.6.0 available from http://subread.sourceforge.net/. All codes used in this study are available at https://github.com/MarioniLab/LncScreen2018.<br>GraphPad Prism6 was used to calculate means, standard deviation, P value and to perform statistical analyses. Fiji Image J was used for imaging analysis.

For manuscripts utilizing custom algorithms or software that are central to the research but not yet described in published literature, software must be made available to editors/reviewers. We strongly encourage code deposition in a community repository (e.g. GitHub). See the Nature Research guidelines for submitting code & software for further information.

Data

Policy information about availability of data

All manuscripts must include a data availability statement. This statement should provide the following information, where applicable:

- Accession codes, unique identifiers, or web links for publicly available datasets
- A list of figures that have associated raw data
- A description of any restrictions on data availability

Sequencing data are available in the ArrayExpress database (http://www.ebi.ac.uk/arrayexpress) with the accession codes E-MTAB-7432 (RNA-seq), E-MTAB-7418 (CHART-seq) and E-MTAB-7419 (CUT&RUN).
The imaging data have been submitted to the Image Data Resource (https://idr.openmicroscopy.org) under IDR accession number idr0056.
Other data used for this study can be found under accessions GSE52139 (Supplementary Figure 12C).

Field-specific reporting

Please select the one below that is the best fit for your research. If you are not sure, read the appropriate sections before making your selection.

[x] Life sciences [ ] Behavioural & social sciences [ ] Ecological, evolutionary & environmental sciences

For a reference copy of the document with all sections, see nature.com/documents/nr-reporting-summary-flat.pdf

Life sciences study design

All studies must disclose on these points even when the disclosure is negative.

Sample size	All experiments were done with at least three biological replicates except the CUT&RUN, which was done with 2 biological replicates. The sample sizes are described in the figure legends.
Data exclusions	No data were excluded.
Replication	All our attempts to replicate the data were successful.
Randomization	No randomization was performed.
Blinding	The experiments were not blinded.

Reporting for specific materials, systems and methods

We require information from authors about some types of materials, experimental systems and methods used in many studies. Here, indicate whether each material, system or method listed is relevant to your study. If you are not sure if a list item applies to your research, read the appropriate section before selecting a response.

Materials & experimental systems

n/a	Involved in the study	
[ ]	[x]	Antibodies
[ ]	[x]	Eukaryotic cell lines
[x]	[ ]	Palaeontology
[x]	[ ]	Animals and other organisms
[x]	[ ]	Human research participants
[x]	[ ]	Clinical data

Methods

n/a	Involved in the study	
[x]	[ ]	ChIP-seq
[x]	[ ]	Flow cytometry
[x]	[ ]	MRI-based neuroimaging

Antibodies

Antibodies used	Antibodies used for Western blotting were TPPP (NBP2-34031, Novus, dilution 1:1000), RAI14 (NBP1-94075, Novus, dilution 1-300), DNAAF5 (HPA020243, Atlas Antibodies, dilution 1-250), ITGB1BP1 (HPA071538, Atlas Antibodies, dilution 1-250), beta-tubulin (T019, Sigma, dilution 1:2000), p150 (610473, BD Transduction Laboratories, dilution 1:2000), cyclin B1 (12231S, Cell signalling, dilution 1:1000) and phospho Histone H3 Serine 10 (06-570, Millipore, dilution 1:1000), Amersham ECL anti-mouse IgG-HRP antibody (NXA931V, GE Healthcare, dilution 1:2000) and Amersham ECL anti-rabbit IgG-HRP antibody (NA934V, GE Healthcare, dilution 1:2000). Antibodies used for immunofluorescence were acetylated alpha-tubulin (T6793, Sigma, dilution 1:500), alpha-tubulin (TUB9026, Sigma, dilution 1:1000), CREST (Antibodies Inc., dilution 1:1000), Mad2 (Biolegend, Babco, dilution 1:100), EB1 (CRUK Hybridoma bank, Zyss et al., 2011, JCB, dilution 1:300), phospho Histone H3 Serine 10 (06-570, Millipore, dilution 1:2000), alpha-tubulin (rat) (MCA78G, AbD Serotec, dilution 1:500), gamma-tubulin (GTU88, Sigma, dilution 1:1000), CDK5RAP2/CEP215 (Barr et al., 2010, JCB, dilution 1:500), Alexa Fluor® 555 goat anti-rat (A-21434, Thermo Fisher Scientific, dilution 1:1000), Alexa Fluor® 488 donkey anti-rabbit (A-21206, Thermo Fisher Scientific, dilution 1:1000), Alexa Fluor® 647 donkey anti-mouse (A-31571, Thermo Fisher Scientific, dilution 1:1000), Alexa Fluor® 647 goat anti-human (A-21445, Thermo Fisher Scientific, dilution 1:1000) and Alexa Fluor 568 Phalloidin (A12380, Thermo Fisher Scientific, dilution 1:500). Antibodies used for CUT&RUN were H3K4me3 (05-1339, Millipore (LOT2780484), dilution 1:100), H3K36me3 (61101, Active Motif (LOT32412003), dilution 1:100), H3K27me3 (9733S, C36B11, Cell Signaling (LOT8), dilution 1:100), H3K27ac (Ab4729, Abcam (LOT GR3187598-1), dilution 1:100), goat anti-rabbit IgG secondary antibody (Ab97047, Abcam (LOT GR254157-8), dilution 1:100) and rabbit anti-mouse IgG secondary antibody (A27022, Thermo Fisher Scientific (LOT RG240909), dilution 1:100).
Validation	All informations about the antibodies used in this study are available in the Supplementary Information.

Eukaryotic cell lines

Policy information about cell lines

Cell line source(s)	All cell lines used in this study were from the ATCC (HeLa, RPE1, MCF10A, HUVEC, 293FT). HeLa Kyoto cells were obtained from ATCC/Jan Ellenberg (EMBL, Heidelberg).
Authentication	All the cell lines were STR profiled to authenticate their identity. The profiles were compared to published profiles and the percent match was calculated using the Tanabe algorithm. We used PowerPlex_16HS_cell line panel and analyzed the results using applied Biosystems gene mapper IDv3.2.1 software by the external provider Genetics DNA Laboratories . The certificates are available upon request.
Mycoplasma contamination	All the cell lines were routinely tested for the mycoplasma contamination and resulted negative. The MycoProbe kit (R&D) was used.
Commonly misidentified lines (See ICLAC register)	No commonly misidentified cell lines were used.
