# Supplementary material for: A high-content RNAi screen reveals multiple roles for long noncoding RNAs in cell division
Source: Nat Commun. 2020 Apr 15;11:1851. doi: 10.1038/s41467-020-14978-7 (PMC7160116; doi:10.1038/s41467-020-14978-7)
Supplement: Supplementary file 4 — Description of Additional Supplementary Files [file 41467_2020_14978_MOESM4_ESM.pdf]

Description of Additional Supplementary Files

File Name: Supplementary Data 1
Description: The list of 2231 lncRNAs in Lincode library.

File Name: Supplementary Data 2
Description: Raw data and the Z-scores for mitotic progression (mitotic index), viability, chromosome segregation and cytokinesis after depletion of 2231 lncRNAs in screen A and B. **1st sheet:** Raw data for screen A and B. **2nd sheet:** Z-scores data for screen A and B.

File Name: Supplementary Data 3
Description: Raw data for the third validation screen (57 lncRNA candidates including Control si (Ambion), GNG12-AS1 (exon 1), Ch-TOG/CKAP5 and ECT2 siRNA). **1st sheet:** single well data for third validation screen. **2nd sheet:** average of single plate data for third validation screen. **3rd sheet:** final plate average for third validation screen.

File Name: Supplementary Data 4
Description: The list of Stellaris FISH probes against linc00899, TPPP and C1QTNF1-AS1. **1st sheet:** Linc00899 RNA FISH exonic probes. **2nd sheet:** C1QTNF1-AS1 RNA FISH exonic probes**. 3rd sheet:** TPPP RNA FISH intronic probes.

File Name: Supplementary Movie 1
Description: Mitosis in HeLa Kyoto cells transfected with control siRNA (Ambion).

File Name: Supplementary Movie 2
Description: Mitosis in HeLa Kyoto cells transfected with linc00899 siRNA

File Name: Supplementary Movie 3
Description: Mitosis in HeLa Kyoto cells transfected with C1QTNF1-AS1 siRNA

File Name: Supplementary Movie 4
Description: Mitosis in HeLa Kyoto cells transfected with control LNA A.

File Name: Supplementary Movie 5
Description: Mitosis in HeLa Kyoto cells transfected with linc00899 LNA1.

File Name: Supplementary Movie 6
Description: Mitosis in HeLa Kyoto cells transfected with C1QTNF1-AS1 LNA1. For all the movies, images were analysed by time-lapse microscopy using Zeiss Axio Observer Z1 microscope. Frames were acquired every 10 min. HeLa Kyoto cells are stably expressing histone H2B-mCheery (red, chromatin marker) and eGFP-a-tubulin (green, microtubule marker).
